# Regulation of *Caenorhabditis elegans* avoidance behavior toward *Pseudomonas aeruginosa* PA14 by the canonical Wnt signaling pathway

**DOI:** 10.3389/fnmol.2026.1851614

**Published:** 2026-08-11

**Authors:** Rong Lin, Keshu Zhao, Qian Zhang, Xuanxue Xiao, Yuping Zhao, Xin Zhao, Wei Zou

**Affiliations:** 1 Zhaotong Health Vocational College, Zhaotong, Yunnan, China; 2 School of Basic Medical Sciences, Kunming Medical University, Kunming, Yunnan, China; 3 Yunnan Provincial Key Laboratory of Public Health and Biosafety and School of Public Health, Kunming Medical University, Kunming, Yunnan, China; 4 Infection Control Office, Xi’an Public Health Center (Xi’an Emergency Medical Center), Xi’an, Shaanxi, China; 5 Yunnan Key Laboratory of Cross-Border Infectious Disease Prevention and New Drug Development, Kunming Medical University, Kunming, Yunnan, China

**Keywords:** Caenorhabditis elegans, npr-1, *Pseudomonas aeruginosa* PA14, Wnt signaling, β-catenin

## Abstract

Using genetic and behavioral analyses, we identified a role for canonical Wnt/β-catenin signaling in the regulation of avoidance behavior toward *Pseudomonas aeruginosa* PA14 in *Caenorhabditis elegans*. RNAi analysis indicated a possible involvement of the Wnt ligand *cwn-2*, although interpretation of *cwn-2* mutant phenotypes was limited by locomotor defects. Genetic and behavioral analyses further implicated the receptor *cfz-2*, the transducer *dsh-1*, and the β-catenin homologue *bar-1* in this behavior, whereas *gsk-3* acted as a negative regulator. Epistasis analyses further supported that *cfz-2* functions genetically upstream of *gsk-3* and *bar-1* in the regulation of avoidance. Reporter analyses showed overlapping expression of *cfz-2* and *dsh-1* in a subset of head-region cells with neuron-like morphology, supporting a possible association between Wnt pathway expression and cells potentially relevant to avoidance-related regulation. However, these data do not establish a neuron-specific site of action. Systems-level analyses further indicated that genes identified through Wnt pathway screening are closely connected to known avoidance-related regulatory networks and are enriched in pathways associated with neuronal development, receptor-mediated signaling, and intercellular communication. Overall, these findings support a role for canonical Wnt/β-catenin signaling in PA14 avoidance and support a potential association between Wnt pathway activity and avoidance-related cellular or regulatory networks.

## 1 Introduction

*Caenorhabditis elegans* (*C. elegans*) has become a useful model organism for investigating how specific genes regulate neural circuits in a relatively simple nervous system and how such regulation is linked to behavioral outputs. In neuroscience research, the use of *C. elegans* in a systems-level framework offers several advantages, including high genetic tractability, a short life cycle, optical transparency, and a fully mapped connectome. These features make it possible to establish functional relationships and conceptual frameworks that can later be tested in higher-order species (Baiocchi et al., 2020; Bhattacharya et al., 2019; Park et al., 2017). With a compact nervous system composed of 302 neurons and a rich repertoire of innate and experience-dependent behaviors, such as chemotaxis, thermotaxis, learning and memory, and pathogen avoidance, *C. elegans* provides a unique and highly controllable platform for quantitatively investigating the molecular and neural mechanisms underlying behavioral decision-making (Rahmani and Chew, 2021; Tsukada and Mori, 2021). A particularly well-established behavioral paradigm for such studies is the bacterial lawn avoidance assay using the pathogenic bacterium *Pseudomonas aeruginosa* PA14. In this assay, nematodes are placed on a lawn of PA14, and pathogen avoidance is defined as the observable behavior in which worms leave the bacterial awn and move to the surrounding bacteria-free area. This assay provides a quantifiable behavioral output for examining how external pathogenic cues are detected, processed by neural circuits, and translated into avoidance-related motor responses (Chevalier et al., 2022; da Cruz Nizer et al., 2021; Meisel and Kim, 2014).

At the molecular level, avoidance behavior toward PA14 has been shown to be precisely regulated by multiple evolutionarily conserved signaling pathways, including p38 MAPK, TGF-β, and insulin/PI3K signaling. These pathways jointly coordinate olfactory and gustatory inputs, oxygen sensing, and the internal metabolic state to shape the final avoidance decision (Aballay, 2009; Chang et al., 2011; Yu et al., 2024). Notably, the neuropeptide receptor *npr-1* pathway has been identified as a central hub in this behavioral regulatory network, dynamically integrating diverse environmental and internal signals to modulate circuit excitability and, consequently, behavioral output. However, behavioral decision-making necessarily involves complex crosstalk among signaling networks and fine spatiotemporal regulation. Whether other key pathways implicated in development and plasticity—particularly the Wnt signaling pathway—participate in real-time pathogen avoidance in the mature nervous system, and if so, how, remains poorly understood.

Wnt signaling is a highly conserved intercellular communication system (Aballay, 2009; Yu et al., 2024). Wnt signaling comprises two fundamental branches: the canonical β-catenin-dependent pathway and the noncanonical pathways, including planar cell polarity (PCP) and Wnt/calcium signaling (Shah et al., 2023). The canonical β-catenin-dependent pathway is triggered when a ligand (e.g., *cwn-2* in *C. elegans*) binds to a Frizzled-family receptor (e.g., *cfz-2*), which initiates a signaling cascade mediated by the Disheveled protein (e.g., *dsh-1*) and inhibits *gsk-3* activity. This action results in the stabilization and nuclear translocation of β-catenin (e.g., *bar-1*), which binds to the TCF/LEF transcription factors and induces the transcription of downstream target genes (Blankesteijn et al., 2008; Shah and Kazi, 2022). The canonical Wnt signaling pathway is critical for embryonic development, tissue homeostasis, stem cell maintenance, and the establishment of synapses. The widely documented developmental functions in *C. elegans*, including the guidance of axons, migration of cells, and establishment of synaptic specificity, support the ability of Wnt signaling to precisely sculpt the connectivity of neural circuits and, therefore, their functions (Ackley, 2014; Zheng et al., 2015).

Cross-species studies have additionally indicated that Wnt/β-catenin signaling is active in adult neurons, wherein it can remodel transcriptional programs involved with receptors/ion channels and neuropeptides, thus affecting the balance between excitation and inhibition and postsynaptic density. Such changes may theoretically decrease the threshold for behavioral responses and increase the input gain (Caracci et al., 2021). In addition, glycogen synthase kinase-3 (*gsk-3*) is positioned at the interface of Wnt signaling and multiple other pathways, including insulin/phosphoinositide 3-kinase (PI3K) and stress-related pathways (e.g., p38), and, therefore, has the potential to coordinate circuit-level regulation of neural function with metabolic and inflammatory pathways (Sai Varshini et al., 2024; Thapa et al., 2023; Zhang et al., 2024). Despite the above evidence, little direct evidence currently exists to indicate that Wnt signaling regulates rapid behavioral flexibility (e.g., pathogen avoidance) in mature animals. It remains to be established how Wnt signaling interacts genetically or functionally with other known master regulators of behavior (e.g., the NPR-1 pathway in *C. elegans*) to achieve coordinated behavioral regulation. These points create a strong biological rationale for the proposed working hypothesis that Wnt signaling is a regulatory contributor to the avoidance of pathogens in adult life.

Accordingly, this study aimed to systematically determine whether the canonical Wnt/β-catenin pathway regulates *C. elegans* avoidance behavior toward PA14 and to define its position within the behavioral regulatory network. We will identify essential pathway components through genetic screening, use reporter-based localization to examine whether key pathway components are expressed in cells potentially relevant to avoidance behavior, and apply protein–protein interaction (PPI) network analysis and Gene Ontology (GO) and Kyoto Encyclopedia of Genes and Genomes (KEGG) enrichment to evaluate the association between this pathway and the neural regulatory network underlying avoidance at a systems level. Finally, through genetic interaction experiments, we will clarify the upstream–downstream relationship between Wnt and core *npr-1* pathways. This study not only provides genetic evidence that canonical Wnt signaling contributes to PA14 avoidance behavior but also offers new insights—through a systems biology lens—into how multiple signaling pathways cooperatively shape behavioral decision-making.

## 2 Materials and methods

### 2.1 Materials

#### 2.1.1 *C. elegans* strains

The wild-type N2 strain was used as the reference wild-type strain in this study. Unless otherwise specified, the terms “wild-type animals” and “wild-type worms” refer to the N2 strain. The mutant strains used in this study included *lin-44*(n1792), *cwn-2*(ok895), *cfz-2*(ok1201), *dsh-1*(ok1445), *npr-1*(ur89), *egl-20*(mu39), *lin-44*(n1792); *egl-20*(n585), and *bar-1*(mu63). All strains were obtained from the Caenorhabditis Genetics Center. The *gsk-3*(tm1237) strain was obtained from the National BioResource Project in Japan. Double mutants *gsk-3*(tm1237); *cfz-2*(ok1201), *gsk-3*(tm1237); *npr-1*(ur89), and *gsk-3*(tm1237); *bar-1*(mu63) were generated by crossing the corresponding single mutants.

*C. elegans* is an invertebrate species that is not currently covered by the jurisdiction of the Institutional Animal Care and Use Committee (IACUC) according to international and national guidelines for laboratory animal ethics. Therefore, ethical approval from the IACUC was not required for this study.

#### 2.1.2 Bacterial strains

The bacterial strains used in this study included *Escherichia coli* OP50, *Pseudomonas aeruginosa* PA14, *E. coli* HT115, and *C. elegans* RNAi feeding clones targeting *cwn-2*, *mom-2*, *dsh-1*, *dsh-2*, *mig-5*, *cfz-2*, *bar-1*, *sys-1*, *wrm-1*, *gsk-3*, *apr-1*, *lrp-1*, *mom-1*, *kin-19*, and *mig-1* (obtained from the *C. elegans* RNAi library of the Yunnan Key Laboratory for Conservation and Utilization of Biological Resources).

#### 2.1.3 Reagents

The reagents used for nematode culture and behavioral assays included agar, peptone, yeast extract, calcium chloride (CaCl_2_), magnesium sulfate (MgSO_4_), dipotassium phosphate, monopotassium phosphate, cholesterol, sodium hydroxide, and anhydrous ethanol. These reagents were used to prepare nematode growth medium (NGM), LB medium, and related buffers for the routine maintenance of *C. elegans*, bacterial cultures, and PA14 avoidance assays. In the preparation of avoidance assay plates, phosphate buffer, MgSO_4_, CaCl_2_, and cholesterol were added to the molten NGM medium before pouring, as described in section 2.2.1. Sodium hypochlorite was used for worm synchronization by bleaching gravid adults when age-matched populations were required for the study. Tris-base was used to prepare routine molecular biology buffers.

The reagents used for RNAi and molecular experiments included IPTG, which was used to induce dsRNA expression in RNAi-feeding bacteria, as described in section 2.2.2. In-Fusion enzyme, PCR amplification reagents, restriction endonucleases (HindIII, KpnI, XbaI, and NaeI), nucleic acid dye, DNA purification kits, and plasmid miniprep kits were used for vector construction, restriction digestion, gel-based fragment verification, DNA recovery, and plasmid preparation during the generation of rescue and fluorescent reporter constructs, as described in section 2.2.3.

Genomic DNA was extracted from worms using animal tissue DNA extraction kits for the PCR-based verification of transgenic lines and mutant genotypes. Total RNA was extracted from worms using RNA extraction kits for quantitative real-time PCR analysis, and cDNA was synthesized using reverse transcription kits prior to qPCR, as described in section 2.2.10 of this manuscript. Unless otherwise indicated, all reagents were of analytical grade and purchased from commercial suppliers, including Sangon Biotech (Shanghai, China), Beijing Biord Biotechnology Co., Ltd. (Beijing, China), Baori Doctor Biotech (Beijing, China), Sigma-Aldrich (Shanghai, China), New England Biolabs (Beijing, China), Axygen Biotechnology (Hangzhou, China), and Beijing Biotech Biotechnology Co., Ltd. (Beijing, China).

#### 2.1.4 Instruments and equipment

Major instruments were used to meet the specific requirements for conducting the experimental procedures. Biochemical incubators and constant-temperature shakers were used to culture and prepare the bacterial cultures of PA14 and RNAi feeding plates. A Laminar Flow Hood was used to provide sterile conditions for handling plates and to conduct the inoculation of bacteria on the plates. Light and fluorescence microscopes were used to conduct routine worm observations and to screen for fluorescently tagged transgenic animals, as well as for imaging GFP/mCherry signals in localization experiments. A micro-injection system was used to provide recombinant plasmids directly into the gonads of adult hermaphrodites in order to generate transgenic lines. Polymerase chain reaction (PCR) thermal cyclers and real-time PCR systems were used for amplification, verification of recombinant constructs, and quantitative analysis of the efficiency of RNAi knockdown. Electrophoresis and gel imaging systems were used to verify PCR products and restriction digestion fragments during vector construction. Refrigerated centrifuges, vortex mixers, ultrapure water systems, and pipettes were used for routine sample preparation and molecular-based experiments.

### 2.2 Methods

#### 2.2.1 Avoidance behavior assay

The avoidance behavior of *C. elegans* toward PA14 was assessed using a previously described lawn avoidance assay with minor modifications (Bai et al., 2021). Briefly, PA14 was revived on LB agar plates, and a single colony was inoculated into LB broth and cultured at 37°C with shaking at 180 rpm for 16 h. After cooling 300 mL of molten NGM medium, 8 mL of phosphate buffer, 300 μL of MgSO_4_, 300 μL of CaCl_2_, and 300 μL of cholesterol solution were added, mixed thoroughly, poured into 6-cm plates, and dried. Then, 20 μL of PA14 culture was spotted at the center of each plate. After drying, the plates were incubated at 37°C for 24 h and then transferred to 25°C for 12 h to prepare PA14 lawns. Non-pathogenic *E. coli* OP50 was used as the control.


AvoidanceIndex=(Numberofwormsoutsidethelawn)/

(T⁢o⁢t⁢a⁢l⁢n⁢u⁢m⁢b⁢e⁢r⁢o⁢f⁢w⁢o⁢r⁢m⁢s)

Synchronized young adult worms were washed with M9 buffer and sterile deionized water, and approximately 30 worms were placed at the center of each bacterial lawn for observation. In this study, avoidance behavior was defined as worms leaving the PA14 bacterial lawn and moving to the surrounding bacteria-free area. The avoidance index was calculated as the proportion of worms that completely left the central lawn area at the indicated time point.

For the initial time-course assay, avoidance indices were measured at 4, 8, 12, 16, and 24 h after the start of the assay. Based on the time-course results and measurement robustness, 8 h was selected as the standard time point for subsequent avoidance assays. A higher avoidance index indicates stronger avoidance behavior toward PA14. To minimize the potential influence of phototaxis on worm movement, all assays were conducted in a light-shielded dark box within a 20°C incubator, without external light exposure during the observation period. For standard avoidance assays, the number of worms inside and outside the lawn was counted after 8 h of incubation at 20°C.

#### 2.2.2 RNAi in *C. elegans*

RNAi bacterial clones targeting genes of interest were streaked onto LB agar plates containing ampicillin and incubated overnight at 37°C. HT115 bacteria carrying an empty vector were used as vector controls. A single colony was inoculated into LB broth containing ampicillin and cultured at 37°C with shaking at 180 rpm for 12 h. An appropriate volume of this culture was then transferred into fresh LB medium and incubated overnight with shaking. The amplified culture was evenly spread onto NGM plates supplemented with IPTG, induced at 37°C for 12 h, and then further induced at 25°C for 12 h to prepare RNAi feeding plates. Finally, synchronized L1 larvae were placed on RNAi plates and cultured at 20°C until the young adult stage for subsequent experiments. This approach is based on RNAi feeding, in which worms ingest bacteria expressing double-stranded RNA to achieve a gene-specific knockdown.

### 2.2.3 Construction of expression vectors and generation of transgenic lines

Relevant expression vectors were constructed in this study to perform functional rescue assays and to analyze fluorescence reporter expression patterns. Rescue constructs, including *Pcfz-2::cfz-2* and *Pbar-1::bar-1*, and fluorescent reporter constructs, including *Pcfz-2::GFP* and *Pdsh-1::mCherry*, were generated using seamless cloning based on homologous recombination.

Genomic sequences of the target genes, including *cfz-2*, *bar-1*, and *dsh-1*, and their predicted promoter regions, approximately 1.5 kb upstream of the start codon ATG, were obtained from WormBase. Gene-specific primers were designed using DNAMAN software (Table 1). Genomic DNA from wild-type N2 worms was used as the template for high-fidelity PCR amplification. Promoter fragments and coding sequences were amplified as required for the corresponding rescue or reporter constructs. The backbone vectors were linearized using restriction enzymes. Specifically, pPD95.75 was used to construct the *cfz-2*- and *bar-1*-related rescue and reporter plasmids and was digested with XbaI and KpnI, whereas pCFJ104 was used to construct the *Pdsh-1::mCherry* reporter plasmid and was digested with XbaI and NaeI. The digested products were separated by agarose gel electrophoresis and purified. Purified PCR fragments were then recombined with the corresponding linearized vectors according to the manufacturer’s instructions for the HD Cloning Kit. The recombinant products were transformed into *E. coli* DH5α competent cells and plated on LB agar containing ampicillin for positive clone selection.

**TABLE 1:** Primer sequences used for vector construction.

Category	Primer name	Sequence (5′→ 3′)
Systemic rescue constructs	*cfz-2* rescue F	GCTTGCATGCCTGCAGGTCGACAGATTCCAGTCAGTTTTCG
	*cfz-2* rescue R	ACTCATTTTTTCTACCGGTACCCAGCTTGTCAGGAATGCCTC
	*bar-1* rescue F	ATGCCTGCAGGTCGACTCTAGAGACAGATTGGACGCTACTG
	*bar-1* rescue R	CTCATTTTTTCTACCGGTACCAAATCGACTATTCCTAGAAGG
Fluorescent localization constructs	*cfz-2* localization F	CATGCCTGCAGGTCGACTCTAGAACGGACCACTACAATGCCAC
	*cfz-2* localization R	TACTCATTTTTTCTACCGGTACCAGCAATAGGAACAGCACGCT
	*dsh-1* localization F	TGTACTCTATCACTGCCGGCGCACCTCGTTCCCTCTTTCA
	*dsh-1* localization R	CAAACTTGTCATTTCTAGAATCATTGTTGTCGGACTCCCG
Sequencing/verification	*cfz-2* verification F	GGTTGGACTCAAGACGATAG
	*cfz-2* verification R	ATTCAAGGGGTGTCAGGT
	*bar-1* verification F	GAAGATGGAGTGTGACCCA
	*bar-1* verification R	AGCACCTTGTTATGCCTGT
	*cfz-2* localization verification F	TGGTTGGAGGTGGTAGAGTCA
	*cfz-2* localization verification R	AGCAATAGGAACAGCACGCT
	*dsh-1* localization verification F	TCTCGCCAGTTTCTTCACCC
	*dsh-1* localization verification R	AGACGACGCAGGGAATGTGT

Single colonies were picked and initially screened by colony PCR using vector-specific universal primers or gene-specific verification primers (Table 1). Positive clones were expanded for plasmid extraction and subjected to restriction enzyme digestion to confirm the expected insert sizes. All candidate plasmids were then verified by bidirectional sequencing using the corresponding verification primers. The resulting sequences were aligned with the expected target sequences to confirm correct insert orientation, an intact reading frame (where applicable), and the absence of unintended mutations. Verified recombinant plasmids were introduced into the gonads of the corresponding mutant worms by microinjection. For injection, the plasmid of interest, at 50–100 ng/μL, was mixed with the co-injection marker plasmid *Pmyo-3::mCherry* at 50 ng/μL. Injected P0 worms were cultured at 20°C, and F1 progeny expressing the red fluorescent marker were selected to establish independent transgenic lines. For each construct, at least two independent lines were generated and stably maintained for subsequent behavioral rescue assays or fluorescence reporter analyses.

#### 2.2.4 Microinjection and transgenic screening

Adult hermaphrodites carrying 1–3 eggs were selected for microinjection under a stereomicroscope. Worms were thoroughly washed with M9 buffer to remove residual bacteria and debris and then transferred onto an agarose pad under an oil layer for immobilization. Injections were performed into the distal syncytial gonad using a pulled glass microinjection needle on an accompanying micromanipulation platform. A pneumatic microinjector was used to deliver stable positive pressure to ensure smooth delivery of the injection solution into the gonads.

After injection, 10 μL of recovery buffer was added to the edge of the slide to release the worms from the oil layer. The worms were then transferred onto NGM plates seeded with OP50 using a worm pick, and an additional 10 μL of recovery buffer was added to facilitate recovery. The plates were incubated at 20°C. For each experiment, 20–30 worms were injected, and a subset of the F1 progeny successfully carried the injected plasmid.

F1 screening was performed using a fluorescence microscope. GFP-positive animals were first identified using a FITC/GFP filter set (green fluorescence). Positive F1 individuals were then continuously cultured at 20°C for approximately 1 week to establish a stably maintained transgenic population. For imaging and behavior-associated observations, adult *cfz-2*::GFP worms carrying 1–3 eggs were selected, washed with M9, and 15 μL of the worm suspension was mounted on slides for image acquisition under the FITC/GFP channel. The experiment was independently repeated thrice, with ≥ 20 worms imaged per replicate.

#### 2.2.5 Genetic crosses and construction of double mutants

Under a stereomicroscope, the sex of *C. elegans* was distinguished based on morphological features: males have a fan-shaped tail with a copulatory bursa and lack a vulva, whereas hermaphrodites have a pointed, slender tail and possess a vulva in the mid-body region. Wild-type N2 males were first crossed with mutant strain A (hermaphrodites) to obtain F1 progeny. Male F1 individuals were crossed with mutant strain B (hermaphrodites) to generate F2 progeny. To facilitate the subsequent isolation of homozygotes, eight F2 hermaphrodites were individually selected and placed on eight separate mating plates for self-fertilization. After each F2 hermaphrodite completed egg-laying, single-worm PCR was performed on the mother to genotype both loci.

If heterozygous bands were observed (both alleles detectable), the mother was judged to be heterozygous at both loci (A/a; B/b) or at least heterozygous at one target locus, and its progeny was retained for homozygote screening; otherwise, the line was discarded. For each plate corresponding to an F2 mother classified as “heterozygous,” 64 hermaphrodite progenies were randomly selected and individually placed onto 64 plates for selfing. After egg laying, single-worm PCR genotyping was conducted for both loci A and B to identify homozygous double mutants (a/a; b/b).

According to Mendelian segregation, the expected frequency of homozygous double mutants was approximately 1/16; therefore, screening ≥ 48–64 individuals per line typically ensured the recovery of positive candidates. Candidate double mutants were confirmed by repeated single-worm PCR and, when feasible, by sequencing; phenotypic validation was performed as needed. Confirmed strains were expanded into stable populations and cryopreserved, and key intermediate cross-generation stages were retained for traceability. To reduce background variation, the final double-mutant strains were backcrossed to the wild-type N2 strain at least three times, or allele-level sequence verification was performed as required by the experimental design.

#### 2.2.6 Fluorescence reporter analysis

The constructed plasmids, *Pcfz-2*::GFP and *Pdsh-1*::mCherry, were diluted in sterile water to a final concentration of approximately 50 ng/μL and mixed in equal ratios to create a co-injection mixture. The mixture was microinjected into the gonadal region of wild-type worms to generate transgenic progeny carrying extrachromosomal arrays. Injected P0 worms were cultured individually at 20°C.

Three days after injection, young adult worms were mounted on slides using 2% agarose pads and examined under a fluorescence microscope. Green and red fluorescence signals were observed using specific excitation/emission channels for GFP (Ex/Em = 488 nm/510 nm) and mCherry (Ex/Em = 587 nm/610 nm), respectively. Images from the two channels were merged using microscope software to assess overlapping reporter expression. Based on fluorescence morphology, cell number, and anatomical position in the head region, the putative identities of expressing cells were tentatively inferred with reference to the *C. elegans* nervous system atlas. Because neuron-specific marker validation was not performed, these assignments were considered tentative and should not be interpreted as evidence for neuron-specific function.

#### 2.2.7 Protein–protein interaction (PPI) network analysis

To explore the potential protein interaction relationships between avoidance-related neuronal genes and the Wnt signaling pathway, we first compiled avoidance-associated neuronal genes reported in the literature and public databases and constructed a candidate gene set based on annotated Wnt pathway components in *C. elegans*. Using Wnt pathway genes as the core, potential interactions between Wnt-related genes and avoidance-associated genes were screened.

Protein–protein interaction information was obtained from the STRING database (Szklarczyk et al., 2023), which includes both known and predicted interactions. Interaction data were imported into Cytoscape for network construction and visualization (Shannon et al., 2003).

#### 2.2.8 Gene ontology (GO) enrichment analysis

GO enrichment analysis of avoidance-related genes identified through Wnt pathway screening was conducted using the clusterProfiler R package. Functional annotation and statistical analyses were performed across three domains: Biological Process (BP), Cellular Component (CC), and Molecular Function (MF). Prior to analysis, gene symbols were converted to Entrez Gene IDs using the org. Ce. eg. db annotation package. Multiple testing correction was performed using the Benjamini–Hochberg method, and the adjusted significance was defined as *P* < 0.05. Enrichment results were visualized using dot plots, in which the point size represents the number of genes enriched in each term, and the color indicates the adjusted significance level.

#### 2.2.9 KEGG pathway enrichment analysis

KEGG pathway enrichment analysis was performed using the clusterProfiler R package with avoidance-related genes screened from the Wnt signaling pathway. *C. elegans* (organism = “cel”) was used as the reference organism, and the input genes were Entrez Gene IDs. Statistical significance was defined as adjusted *P* < 0.05. The results are presented as dot plots, where the point size represents the number of genes involved in a given pathway and the color represents the adjusted *P*-value, enabling the assessment of the functional distribution of avoidance-related neuronal genes across signaling pathways.

#### 2.2.10 Quantitative real-time PCR (qPCR)

Worms subjected to RNAi were cultured to the L4 stage for all genes examined in this study. Approximately 1,000 worms were collected from each RNAi treatment group and from the HT115 empty-vector control group. Total RNA was extracted using an RNA extraction kit (AXYGEN), and 1 μg of total RNA was reverse-transcribed into cDNA using a reverse transcription kit (Takara), according to the manufacturers’ instructions. Quantitative PCR was then performed using gene-specific primers listed in Table 2, with actin-1 as the internal reference gene.

**TABLE 2:** Primer sequences used for quantitative real-time PCR (qPCR).

Gene	Forward primer (5′→3′)	Reverse primer (5′→3′)
*actin-1*	GGATTCTGCAGAAACTCGGA	CATCCAGATCCGCCATGT
*cwn-2*	AACAAGAGCATTGCCAACG	TATCCTCGTCCACAGCACA
*mom-2*	AGCGGATGCTTGGTGGTTAC	TTCGCTTCTGTTGAGGCGTT
*cfz-2*	TGTGCATTTTGGGATGGACG	AGATCGGGAGGAACCCAAAC
*mig-1*	GTCTCGAGATGCTCCACTCA	TGAGCAAGTTGCCGTGTAGT
*dsh-1*	TGGCTCCGTCAATGGTAAGTG	TTGCTGCAATTGAGCTGGTG
*dsh-2*	ATGGCGTCAACAGCAAAAGG	AGACCCATTGACGATTCGGT
*mig-5*	CATCCTCCAAAAGGCAGAACA	TCGTAAAGCATTCGCTCCTCA
*gsk-3*	TCTATTCGAGCGGCGAGAAG	TAGTGCCGGGCAACTCTGTA
*bar-1*	AAGGTGTCCGCGTGAAAGAT	TGCAGGAAGAAAACAACCTCG
*sys-1*	CAAGCGGCTGAACAAGCAAT	TGTTGTACGCAGTTCGGCAT
*wrm-1*	TTCCTCAACGGACCTGCAAC	GTGCATGGTTGCGAGAATGT
*lrp-1*	ATCCACGTCAGCATCTGCAA	TGGCTTCGAGATTTCCGCTT
*apr-1*	TTTCGCGAAGATACGGCGAT	TCATCTTCATCGGTCGGAACA
*mom-1*	ATGTGTGTTGCTGCCAAAGC	GCACGTCCTGTTTTGTTTGAGT
*kin-19*	AGGAGGTGGCCATTAAGCTG	CTCGGTTCCGTACCATCTGA

For each biological replicate, qPCR reactions were performed in three technical replicate wells, and the mean Ct value of the technical replicates was used for subsequent analysis. Relative mRNA expression levels were calculated using the 2^-ΔΔCt method, where ΔCt = Ct (target gene) - Ct(actin-1), and ΔΔCt = ΔCt (RNAi group) - ΔCt (vector control group). Relative expression in the vector control group was set to 1.

For qPCR-based validation of RNAi efficiency, three independent biological replicates were analyzed for each condition. Primer specificity was verified by melt-curve analysis, which showed a single peak for each primer pair. In addition, the amplification efficiency for each primer pair was evaluated using serially diluted cDNA, and only primers with acceptable efficiency were used for relative quantification.

#### 2.2.11 Body-bends assay

Worms were raised under standard laboratory conditions (20 °C on OP50-seeded NGM plates) until they reached the young adult stage, at which time they were moved to unseeded NGM plates for 10–15 min to allow for appropriate acclimation, which was necessary for the removal of residual bacteria from their external surfaces. A single worm was then placed into a 60 μL droplet of M9 buffer on a glass microscope slide surface. The worms were then allowed to acclimate for 30 s before their swimming behavior was recorded for 60 s using an upright microscope with a 40 × objective lens.

Bending frequency was considered as one bending movement if the worm’s head bent from the left (maximum bending) position to the next left (maximum bending) position (same side) and each time the worm’s head moved from the last left (maximum bending) position to the right (maximum bending) position, this was counted as one bend. If there was a bend in the worm’s body that was a consequence of bumping into the edge of the liquid droplet or making a sudden turn that caused the worm to change direction, these movements were excluded from the analysis.

Each treatment group was assessed with three biological replicates and a total of 30 worms per biological replicate. Data are presented as (mean ± standard deviation data) the average number of bends recorded per 60 s. For body-bends assays, each dot represents an individual worm, and the horizontal line indicates the median. Data were summarized as median with interquartile range (IQR). Comparisons between two groups were performed using the two-tailed Mann–Whitney U test, and comparisons among multiple groups were performed using the Kruskal–Wallis test followed by Dunn’s multiple-comparisons test. A value of *P* < 0.05 was considered statistically significant.

### 2.2.12 Morphological assessment and body-size measurements

To assess whether Wnt pathway perturbation caused gross developmental or morphological defects under the experimental conditions used in this study, body size was measured in RNAi-treated animals and mutant strains used for PA14 avoidance assays. Synchronized animals were grown under the same conditions as those used for the corresponding behavioral experiments. At the young adult stage, worms were imaged using a light microscope. Body width and body length were measured from bright-field images using ImageJ software. Body width was measured at the widest region of the body, and body length was measured along the longitudinal body axis from the head to the tail. For each group, 30 animals were analyzed. Gross morphological abnormalities, including obvious body shape changes, developmental delay, and visible head or tail defects, were also inspected during image acquisition. Data are presented as mean ± SD, and statistical comparisons between two groups were performed using a two-tailed unpaired Student’s *t*-test.

#### 2.2.13 Statistical analysis

Statistical analyses were performed using GraphPad Prism 8.4.0 and SPSS 21.0. For qPCR, PA14 avoidance assays, and body size measurements, data are presented as mean ± SD. For qPCR experiments, biological replicates represented independent worm samples collected from separately prepared treatment groups. For PA14 avoidance assays, one biological replicate was defined as one independent experiment performed with a distinct batch of synchronized worms; each biological replicate contained three technical replicate plates, and the mean value of the technical replicates was used for statistical analysis. For qPCR, PA14 avoidance assays, and body size measurements, comparisons between two groups were performed using a two-tailed unpaired Student’s *t*-test, whereas comparisons among three or more groups were performed using one-way ANOVA followed by Tukey’s multiple-comparisons test. For body-bend assays, data are presented as median with interquartile range (IQR), and each dot represents an individual worm. Comparisons between two groups were performed using the two-tailed Mann–Whitney U test, whereas comparisons among three or more groups were performed using the Kruskal–Wallis test, followed by Dunn’s multiple-comparisons test. Statistical significance is indicated as ns, *P* ≥ 0.05; *P < 0.05; **P < 0.01; ***P < 0.001.

## 3 Results

### 3.1 RNAi analysis suggests a possible role for the Wnt ligand *cwn-2* in PA14 avoidance

**FIGURE 1:**
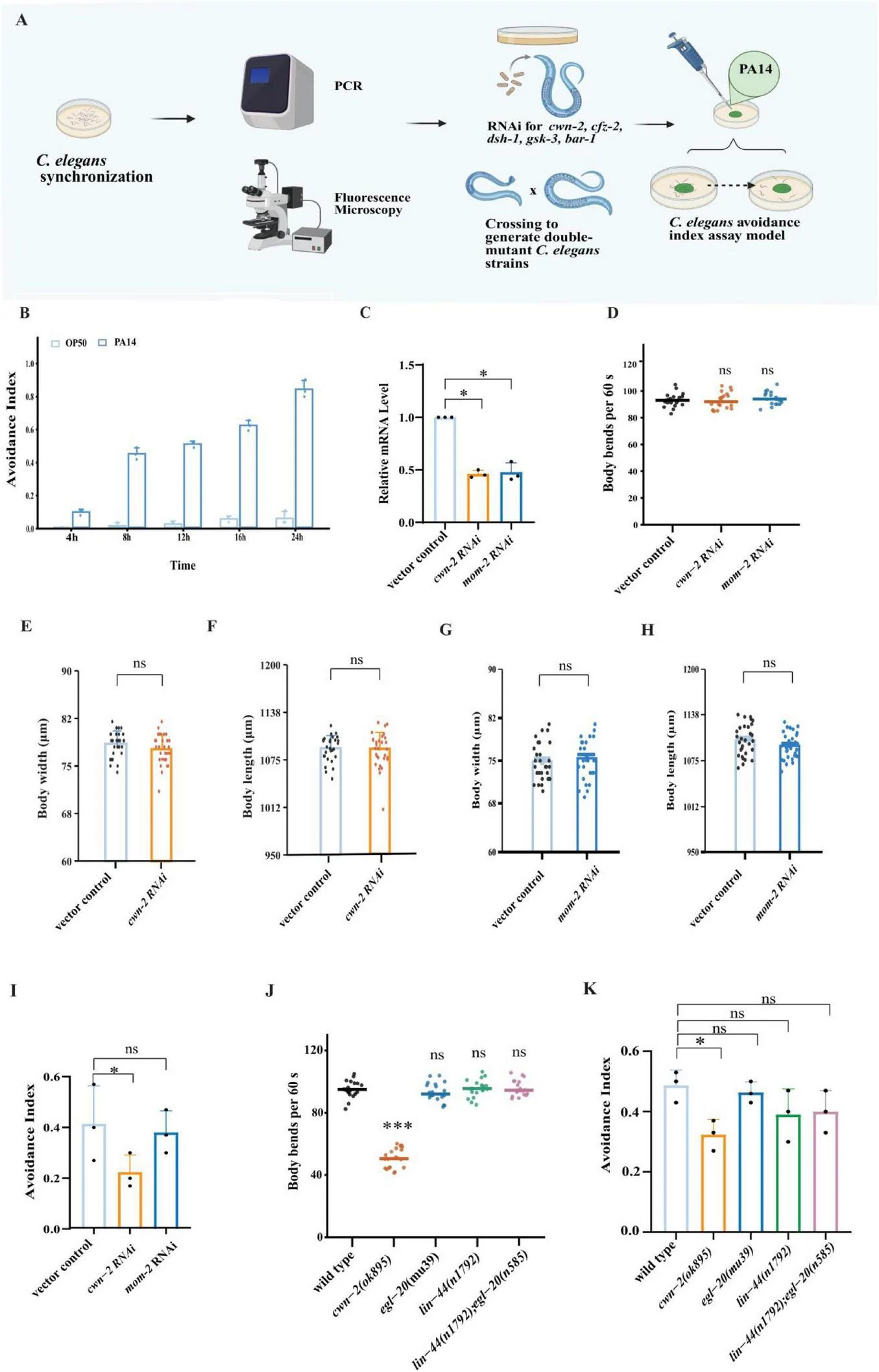
The Wnt ligand cwn-2 contributes to pathogen avoidance behavior in *Caenorhabditis elegans*. **(A)** Schematic overview of the experimental workflow, including *C. elegans* synchronization, PCR-based strain validation, RNAi treatment targeting Wnt pathway components, generation of double-mutant strains, fluorescence microscopy, and PA14 avoidance assays. **(B)** Time course of pathogen avoidance in wild-type animals exposed to OP50 or PA14. Avoidance behavior was quantified as the avoidance index, defined as the fraction of animals outside the bacterial lawn at the indicated time points. **(C)** qPCR validation of RNAi efficiency for *cwn-2* and *mom-2*. Relative mRNA levels were normalized to the vector control. **(D)** Body-bend assay of vector control, *cwn-2* RNAi, and *mom-2* RNAi animals. Body bends were quantified over 60 s. **(E,F)** Body-width **(E)** and body-length **(F)** measurements of vector control and *cwn-2* RNAi animals. **(G,H)** Body-width **(G)** and body-length **(H)** measurements of vector control and *mom-2* RNAi animals. **(I)** PA14 avoidance in vector control, *cwn-2* RNAi, and *mom-2* RNAi animals. Avoidance behavior was quantified as the avoidance index. **(J)** Body-bend assay of wild-type animals and the indicated Wnt ligand mutants, including *cwn-2*(ok895), *egl-20*(mu39), *lin-44*(n1792), and *lin-44*(n1792)*; egl-20*(n585). Body bends were quantified over 60 s. **(K)** PA14 avoidance in wild-type animals and the indicated Wnt ligand mutants. Avoidance behavior was quantified as the avoidance index. Data are presented as mean ± SD. For qPCR experiments, biological replicates represented independent worm samples collected from separately prepared treatment groups. For body-size measurements, 30 animals per group were analyzed. For body-bend assays, individual animals were quantified over 60 s. For avoidance assays, each biological replicate represented one independent experiment performed with a distinct batch of synchronized worms; each biological replicate contained three technical replicate plates, and the mean of the technical replicates was used for statistical analysis. Statistical comparisons for avoidance assays were performed within matched experimental batches using the corresponding vector control or wild-type control included in the same experiment. Statistical significance was determined using one-way ANOVA followed by Tukey’s multiple-comparisons test, or a two-tailed unpaired Student’s *t*-test where appropriate. ns, *P* ≥ 0.05; *P < 0.05; ***P < 0.001.

To establish the PA14 avoidance assay, we first examined the time course of avoidance in wild-type animals exposed to either non-pathogenic OP50 or pathogenic PA14 (Figures 1A,B). Wild-type animals showed minimal avoidance of OP50 lawns, with avoidance indices of 0, 0.01 ± 0.02, 0.01 ± 0.02, 0.05 ± 0.02, and 0.05 ± 0.04 at 4, 8, 12, 16, and 24 h, respectively. In contrast, PA14 exposure induced a time-dependent increase in avoidance, with avoidance indices of 0.09 ± 0.02, 0.43 ± 0.04, 0.49 ± 0.02, 0.60 ± 0.02, and 0.81 ± 0.05 at the corresponding time points. A pronounced avoidance response was evident at 8 h, and the avoidance index continued to increase with prolonged exposure. These results are consistent with previous findings showing progressive avoidance of PA14 lawns by *C. elegans* (Bai et al., 2021). Therefore, 8 h was selected as the standard time point for subsequent avoidance assays.

To investigate whether Wnt ligands contribute to PA14 avoidance, we performed an RNAi-based screen targeting Wnt ligand genes. qPCR analysis confirmed efficient knockdown of *cwn-2* and *mom-2*, with relative mRNA levels reduced to 0.46 ± 0.04 and 0.48 ± 0.09, respectively, compared with the vector control group (both *P* < 0.05; Figure 1C). Because Wnt signaling is associated with developmental and locomotor-related processes (Özhan et al., 2013), we assessed whether RNAi treatment affected basal locomotor activity. Body-bend assays showed that worms treated with *cwn-2* RNAi and *mom-2* RNAi displayed body-bend frequencies of 92 and 94 bends/min, respectively, with no significant difference compared with the vector control group (Figure 1D).

We also assessed gross morphology and body size in *cwn-2* and *mom-2* RNAi animals. *cwn-2* RNAi did not significantly alter body width or body length compared with vector control animals (body width: 77.63 ± 2.34 vs. 78.50 ± 2.03 μm; body length: 1088.00 ± 23.11 vs. 1090.30 ± 16.59 μm; both *P* ≥ 0.05; Figures 1E,F). Similarly, *mom-2* RNAi did not significantly affect body width or body length compared with vector control animals (body width: 75.40 ± 3.33 vs. 74.83 ± 3.06 μm, *P* = 0.495; body length: 1095.33 ± 17.83 vs. 1102.97 ± 21.28 μm, *P* = 0.138; Figures 1G,H). No obvious developmental delay, body shape abnormalities, or visible head/tail defects were observed in either RNAi condition under the conditions used for the avoidance assay. In the PA14 avoidance assay, *cwn-2* RNAi significantly reduced the avoidance index compared with the vector control group (0.22 ± 0.07 vs. 0.48 ± 0.09, *P* < 0.05), whereas *mom-2* RNAi did not produce a significant effect (0.38 ± 0.09; Figure 1I).

To further examine the possible involvement of *cwn-2*, we analyzed locomotor activity and PA14 avoidance in *cwn-2*(ok895) and other Wnt ligand mutant animals, including *lin-44*(n1792), *egl-20*(mu39), and *lin-44*(n1792); *egl-20*(n585) (Figures 1J,K). These mutants were included as available Wnt ligand mutant strains to explore whether the disruption of Wnt ligands other than *cwn-2* also affected PA14 avoidance. The *cwn-2*(ok895) mutant showed a markedly reduced body-bend frequency compared with wild-type animals (68 vs. 113 bends/min; Figure 1J). In contrast, the other Wnt ligand mutants did not exhibit significant locomotor defects. In the avoidance assay, *cwn-2*(ok895) animals showed an avoidance index of 0.32 ± 0.05, whereas the other Wnt ligand mutants showed avoidance indices of 0.46 ± 0.04, 0.39 ± 0.09, and 0.40 ± 0.07 (Figure 1K). However, because *cwn-2*(ok895) animals also displayed reduced body bend frequency, this behavioral phenotype cannot be directly attributed to a specific role of *cwn-2* in PA14 avoidance regulation.

Together, the RNAi data support that reduced *cwn-2* expression may impair PA14 avoidance without affecting basal locomotion or gross body size under the RNAi conditions tested. However, the interpretation of *cwn-2* mutant phenotypes is complicated by the reduced locomotor ability observed in *cwn-2*(ok895) animals. Therefore, although *cwn-2* may be involved in PA14 avoidance, its specific contribution to avoidance regulation cannot be conclusively separated from locomotor effects based on the current mutant data.

### 3.2 The Wnt receptor *cfz-2* regulates *C. elegans* avoidance of PA14

**FIGURE 2:**
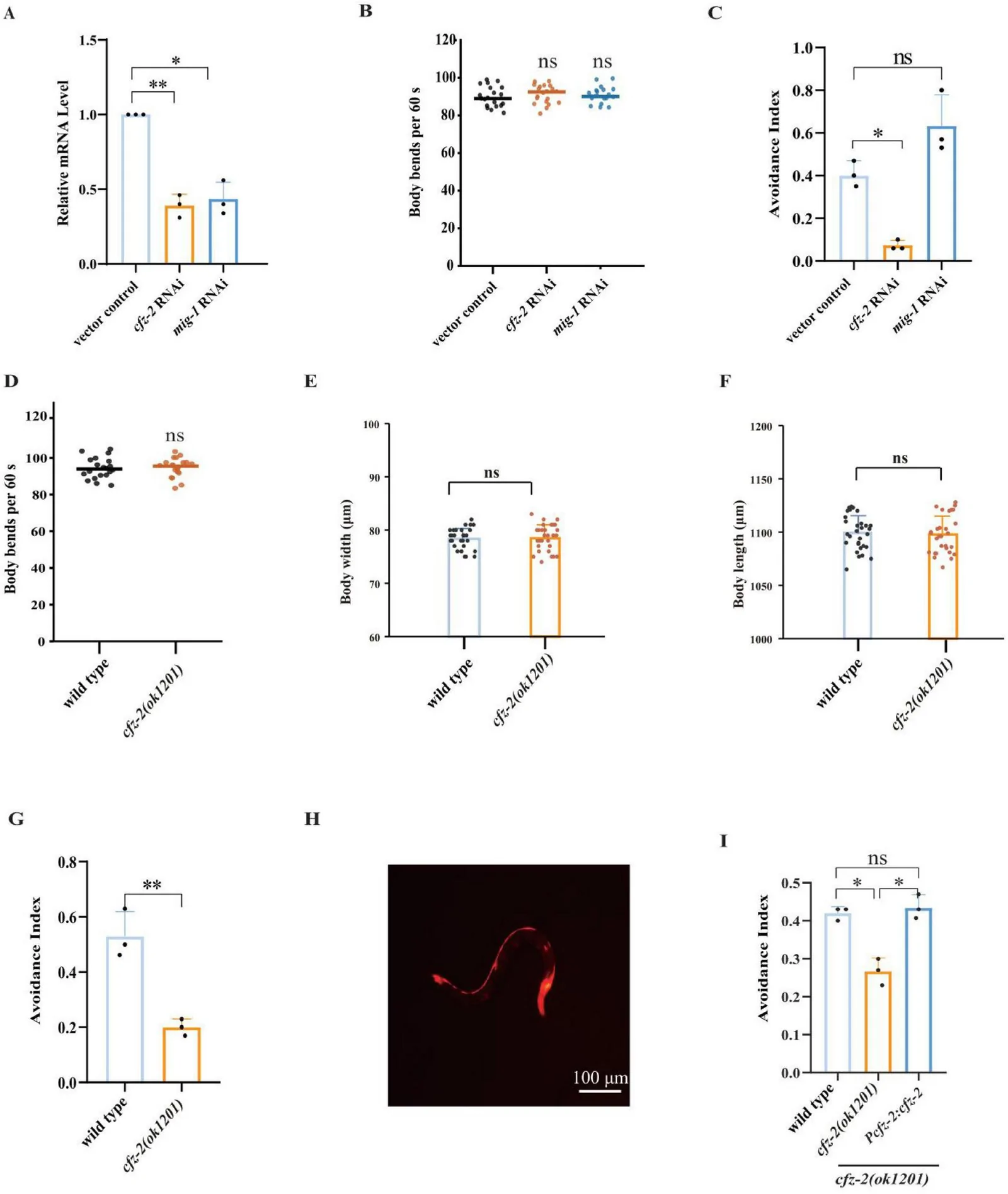
The Wnt receptor ***cfz-2*** contributes to pathogen avoidance behavior in *Caenorhabditis elegans*. **(A)** qPCR validation of RNAi efficiency for *cfz-2* and *mig-1*. Relative mRNA levels are shown after normalization to the vector control. **(B)** Body-bend assay of vector control, *cfz-2* RNAi, and *mig-1* RNAi animals. Body bends were quantified over 60 s. **(C)** PA14 avoidance in vector control, *cfz-2* RNAi, and *mig-1* RNAi animals. Avoidance behavior was quantified as the avoidance index, defined as the fraction of animals outside the PA14 lawn after 8 h. **(D)** Body-bend assay of wild-type animals and *cfz-2*(ok1201) animals. Body bends were quantified over 60 s. **(E)** Body width measurement of wild-type animals and *cfz-2*(ok1201) animals. **(F)** Body length measurement of wild-type animals and *cfz-2*(ok1201) animals. **(G)** PA14 avoidance in wild-type animals and *cfz-2*(ok1201) animals. Avoidance behavior was quantified as the avoidance index. **(H)** Representative fluorescence image of a transgenic *cfz-2*(ok1201) animal carrying a red fluorescent co-injection marker. Scale bar, 100 μm. **(I)** Rescue of the avoidance defect in *cfz-2*(ok1201) animals by expression of wild-type *cfz-2* under its endogenous promoter (*Pcfz-2::cfz-2*). Avoidance behavior was quantified as the avoidance index. Data are presented as mean ± SD. For qPCR experiments, biological replicates represented independent worm samples collected from separately prepared treatment groups. For body-bend assays, each group included three biological replicates with 30 worms per replicate. For body-size measurements, 30 animals per group were analyzed. For avoidance assays, each biological replicate represented one independent experiment performed with a distinct batch of synchronized worms; each biological replicate contained three technical replicate plates, and the mean of the technical replicates was used for statistical analysis. Statistical comparisons for avoidance assays were performed within matched experimental batches using the corresponding vector control or wild-type control included in the same experiment. Statistical significance in **(A–C,I)** was determined using one-way ANOVA followed by Tukey’s multiple-comparisons test. Statistical significance in **(D–G)** was determined using a two-tailed unpaired Student’s *t*-test. ns, *P* ≥ 0.05; *P < 0.05; **P < 0.01; ***P < 0.001.

Because the ligand RNAi screen supported a possible involvement of Wnt signaling in PA14 avoidance, we systematically examined whether Wnt receptors contribute to this behavior. RNAi knockdown of the receptor genes *cfz-2* and *mig-1* was first performed. qPCR validation confirmed effective gene knockdown, with the mRNA levels of *cfz-2* and *mig-1* significantly reduced to 0.39 ± 0.07 and 0.43 ± 0.11, respectively, compared with the vector control (both *P* < 0.05; Figure 2A). Body-bend assays showed that worms treated with *cfz-2* RNAi and *mig-1* RNAi had locomotor frequencies of 92.5 bends/min and 90 bends/min, respectively, with no significant differences compared with the vector control (Figure 2B). These results indicate that RNAi knockdown of *cfz-2* or *mig-1* did not impair basal locomotor ability.

We measured PA14 avoidance after RNAi treatment. Compared with the vector control, *cfz-2* RNAi significantly reduced the avoidance index (0.07 ± 0.02 vs. 0.40 ± 0.07, *P* < 0.05), whereas *mig-1* RNAi did not significantly affect avoidance behavior (0.63 ± 0.15, *P* > 0.05; Figure 2C). To validate the role of *cfz-2* genetically, we further examined the *cfz-2*(ok1201) mutant. Body-bend assays showed that the locomotor frequency of *cfz-2*(ok1201) animals was not significantly different from that of wild-type animals (96 vs. 94.5 bends/min; Figure 2D). In addition, *cfz-2*(ok1201) mutants did not show significant gross body-size abnormalities compared with wild-type animals, with no significant differences in body width or body length (body width: 78.47 ± 2.50 vs. 78.33 ± 1.95 μm; body length: 1097.67 ± 17.36 vs. 1099.27 ± 16.13 μm; both *P* ≥ 0.05; Figures 2E,F). In contrast, the *cfz-2*(ok1201) mutant exhibited a significantly reduced avoidance index compared with wild-type controls (0.20 ± 0.03 vs. 0.53 ± 0.09, *P* < 0.01; Figure 2G), supporting a role for *cfz-2* in PA14 avoidance.

To confirm the specificity of the *cfz-2* phenotype, we examined whether the expression of wild-type *cfz-2* under its endogenous promoter could rescue the reduced avoidance phenotype of *cfz-2*(ok1201) animals (Figures 2H,I). In this matched experimental set, *cfz-2*(ok1201) mutants exhibited a lower avoidance index than wild-type controls (0.27 ± 0.04 vs. 0.42 ± 0.02, *P* < 0.05). Reintroduction of the P*cfz-2*::*cfz-2* transgene restored the avoidance index to 0.43 ± 0.04, which was comparable to that of wild-type animals (*P* > 0.05 vs. wild-type animals; Figure 2I). Collectively, these results indicate that the Wnt receptor *cfz-2* is required for normal avoidance behavior of *C. elegans* toward PA14.

### 3.3 The Disheveled family member *dsh-1* regulates *C. elegans* avoidance of PA14

**FIGURE 3:**
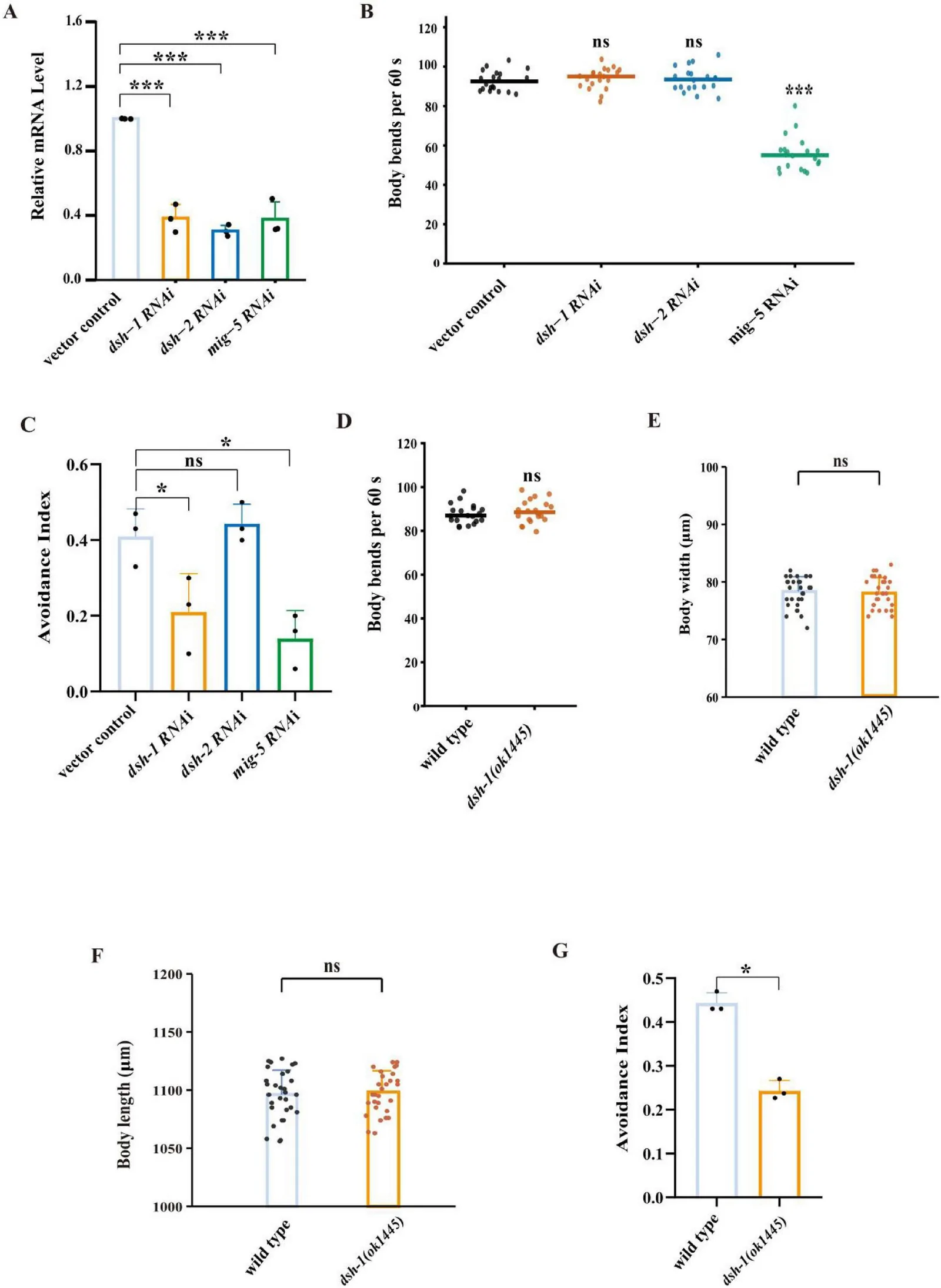
The Disheveled ortholog ***dsh-1*** contributes to pathogen avoidance behavior in *Caenorhabditis elegans*. **(A)** qPCR validation of RNAi efficiency for *dsh-1*, *dsh-2*, and *mig-5*. Relative mRNA levels are shown after normalization to the vector control. **(B)** Body-bend assay of vector control, *dsh-1* RNAi, *dsh-2* RNAi, and *mig-5* RNAi animals. Body bends were quantified over 60 s. **(C)** PA14 avoidance in vector control, *dsh-1* RNAi, *dsh-2* RNAi, and *mig-5* RNAi animals. Avoidance behavior was quantified as the avoidance index, defined as the fraction of animals outside the PA14 lawn after 8 h. **(D)** Body-bend assay of wild-type animals and *dsh-1*(ok1445) animals. Body bends were quantified over 60 s. **(E)** Body width measurement of wild-type animals and *dsh-1*(ok1445) animals. **(F)** Body length measurement of wild-type animals and *dsh-1*(ok1445) animals. **(G)** PA14 avoidance in wild-type animals and *dsh-1*(ok1445) animals. Avoidance behavior was quantified as the avoidance index. Data are presented as mean ± SD. For qPCR experiments, biological replicates represented independent worm samples collected from separately prepared treatment groups. For body-bend assays, each group included three biological replicates with 30 worms per replicate. For body-size measurements, 30 animals per group were analyzed. For avoidance assays, each biological replicate represented one independent experiment performed with a distinct batch of synchronized worms; each biological replicate contained three technical replicate plates, and the mean of the technical replicates was used for statistical analysis. Statistical comparisons for avoidance assays were performed within matched experimental batches using the corresponding vector control or wild-type control included in the same experiment. Statistical significance in **(A–C)** was determined using one-way ANOVA followed by Tukey’s multiple-comparisons test. Statistical significance in **(D–G)** was determined using a two-tailed unpaired Student’s *t*-test. ns, *P* ≥ 0.05; *P < 0.05; **P < 0.01; ***P < 0.001.

Next, we examined the potential roles of the three Disheveled family genes, dsh-1, dsh-2, and mig-5, in PA14 avoidance. qPCR analysis confirmed effective RNAi knockdown, with the relative mRNA levels of dsh-1, dsh-2, and mig-5 reduced to 0.38 ± 0.09, 0.30 ± 0.04, and 0.38 ± 0.11, respectively, compared with the vector control (Figure 3A). Body-bend assays showed that worms treated with dsh-1 and dsh-2 RNAi displayed body-bend frequencies of 95 bends/min and 93.5 bends/min, respectively, which were not significantly different from the vector control (92.5 bends/min; Figure 3B). In contrast, mig-5 RNAi significantly reduced body-bend frequency to 55 bends/min, indicating that mig-5 knockdown impaired basal locomotor activity (Figure 3B).

In the PA14 avoidance assay, dsh-1 RNAi and mig-5 RNAi significantly reduced the avoidance index compared with the vector control (0.21 ± 0.10 and 0.14 ± 0.07 vs. 0.41 ± 0.07, respectively; both *P* < 0.05), whereas dsh-2 RNAi did not significantly affect avoidance behavior (0.44 ± 0.05, *P* > 0.05; Figure 3C). Because mig-5 RNAi also impaired locomotor activity, the reduced avoidance index observed after mig-5 knockdown may be partly attributable to locomotor defects. Therefore, mig-5 was not further pursued in subsequent analyses.

To further examine the role of dsh-1, we analyzed the dsh-1(ok1445) mutant. Body-bend assays showed that the locomotor frequency of dsh-1(ok1445) animals was not significantly different from that of wild-type animals (93 vs. 87 bends/min; Figure 3D), indicating that this mutant did not exhibit a detectable basal locomotor defect. In addition, dsh-1(ok1445) animals did not show significant gross body-size abnormalities compared with wild-type animals, with no significant differences in body width or body length (body width: 78.07 ± 2.69 vs. 78.33 ± 2.58 μm; body length: 1098.37 ± 18.28 vs. 1096.17 ± 20.98 μm; both *P* ≥ 0.05; Figures 3E,F). In contrast, dsh-1(ok1445) animals exhibited a lower avoidance index than wild-type controls (0.24 ± 0.02 vs. 0.44 ± 0.02, *P* < 0.05; Figure 3G). These results support a role for the Disheveled family member dsh-1 in the regulation of *C. elegans* avoidance behavior toward PA14.

### 3.4 The glycogen synthase kinase-3 gene *gsk-3* negatively regulates *C. elegans* avoidance of *Pseudomonas aeruginosa* PA14

**FIGURE 4:**
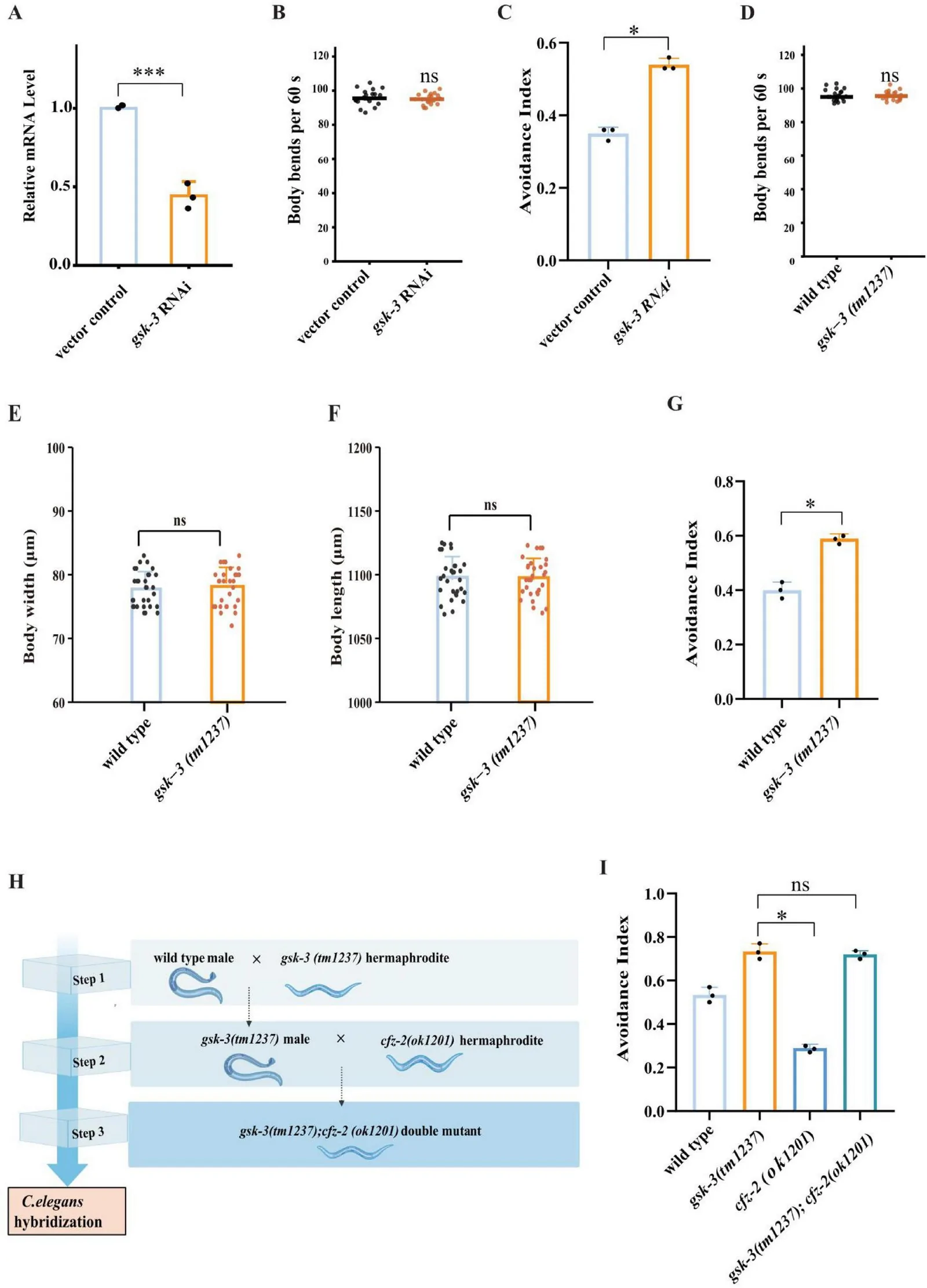
*gsk-3* negatively regulates pathogen avoidance and functions genetically downstream of the receptor ***cfz-2*** in *Caenorhabditis elegans*. **(A)** qPCR validation of *gsk-3* RNAi efficiency. Relative *gsk-3* mRNA levels are shown after normalization to the vector control. **(B)** Body-bend assay of vector control and *gsk-3* RNAi animals. Body bends were quantified over 60 s. **(C)** PA14 avoidance in vector control and *gsk-3* RNAi animals. Avoidance behavior was quantified as the avoidance index, defined as the fraction of animals outside the PA14 lawn after 8 h. **(D)** Body-bend assay of wild-type animals and *gsk-3*(tm1237) animals. Body bends were quantified over 60 s. **(E)** Body width measurement of wild-type animals and *gsk-3*(tm1237) animals. **(F)** Body length measurement of wild-type animals and *gsk-3*(tm1237) animals. **(G)** PA14 avoidance in wild-type animals and *gsk-3*(tm1237) animals. Avoidance behavior was quantified as the avoidance index. **(H)** Simplified schematic of the crossing strategy used to generate the *gsk-3*(tm1237)*; cfz-2*(ok1201) double mutant. Male and hermaphrodite animals are labeled to clarify the parental animals used in each cross, and homozygous double mutants were identified by PCR genotyping. **(I)** Epistasis analysis of *gsk-3* and *cfz-2*. Avoidance behavior was assessed in wild-type animals, *gsk-3*(tm1237), *cfz-2*(ok1201), and *gsk-3*(tm1237)*; cfz-2*(ok1201) animals. Avoidance behavior was quantified as the avoidance index. The double mutant showed an avoidance phenotype comparable to that of the *gsk-3* single mutant, placing *gsk-3* genetically downstream of *cfz-2* in the regulation of PA14 avoidance. Data are presented as mean ± SD. For qPCR experiments, biological replicates represented independent worm samples collected from separately prepared treatment groups. For body-bend assays, each group included three biological replicates with 30 worms per replicate. For body-size measurements, 30 animals per group were analyzed. For avoidance assays, each biological replicate represented one independent experiment performed with a distinct batch of synchronized worms; each biological replicate contained three technical replicate plates, and the mean of the technical replicates was used for statistical analysis. Statistical comparisons for avoidance assays were performed within matched experimental batches using the corresponding vector control or wild-type control included in the same experiment. Statistical significance in **(A–G)** was determined using a two-tailed unpaired Student’s *t*-test. Statistical significance in **(I)** was determined using one-way ANOVA followed by Tukey’s multiple-comparisons test. ns, *P* ≥ 0.05; *P < 0.05; **P < 0.01; ***P < 0.001.

To further examine the role of canonical Wnt signaling in PA14 avoidance, we analyzed gsk-3, which encodes the *C. elegans* homologue of glycogen synthase kinase-3. RNAi-mediated knockdown of gsk-3 significantly reduced its mRNA level to 0.44 ± 0.09 relative to the vector control, confirming effective gene silencing (Figure 4A). Body-bend assays showed that gsk-3 RNAi did not significantly affect basal locomotor ability, with body-bend frequencies of 95.5 bends/min in gsk-3 RNAi animals and 95 bends/min in worms treated with the vector control (Figure 4B). In the PA14 avoidance assay, gsk-3 RNAi animals exhibited a significantly higher avoidance index than worms treated with the vector control (0.54 ± 0.02 vs. 0.35 ± 0.02, *P* < 0.05; Figure 4C), suggesting that gsk-3 negatively regulates PA14 avoidance.

To validate this result genetically, we examined the gsk-3(tm1237) mutant. The locomotor frequency of gsk-3(tm1237) animals was 95.5 bends/min, which was not significantly different from that of wild-type animals (95 bends/min; Figure 4D), indicating that the mutant did not show a detectable basal locomotor defect. In addition, gsk-3(tm1237) animals did not show significant gross body-size abnormalities compared with wild-type animals, with no significant differences in body width or body length (body width: 78.17 ± 3.01 vs. 77.77 ± 2.74 μm; body length: 1097.70 ± 15.14 vs. 1097.93 ± 16.30 μm; both *P* ≥ 0.05; Figures 4E,F). In the avoidance assay, gsk-3(tm1237) animals showed a significantly higher avoidance index than wild-type animals within the same matched experimental set (0.59 ± 0.02 vs. 0.40 ± 0.03, *P* < 0.05; Figure 4G). Together with the RNAi result, this phenotype supports a negative regulatory role for gsk-3 in PA14 avoidance under matched assay conditions.

To determine whether gsk-3 functions downstream of the Wnt receptor cfz-2, we analyzed the gsk-3(tm1237); cfz-2(ok1201) double mutant (Figure 4H). The avoidance index of the double mutant was not significantly different from that of the gsk-3(tm1237) single mutant (0.72 ± 0.02 vs. 0.73 ± 0.04, *P* > 0.05), and both were significantly higher than that of wild-type animals (0.53 ± 0.04, *P* < 0.05). In contrast, the cfz-2(ok1201) single mutant showed a significantly lower avoidance index than wild-type animals (0.29 ± 0.02, *P* < 0.05; Figure 4I). Within this matched experimental set, the gsk-3(tm1237); cfz-2(ok1201) double mutant resembled the gsk-3(tm1237) single mutant rather than the cfz-2(ok1201) single mutant, supporting the placement of gsk-3 genetically downstream of cfz-2 in the regulation of PA14 avoidance.

### 3.5 The β-catenin homolog *bar-1* acts downstream of *gsk-3* to promote PA14 avoidance

**FIGURE 5:**
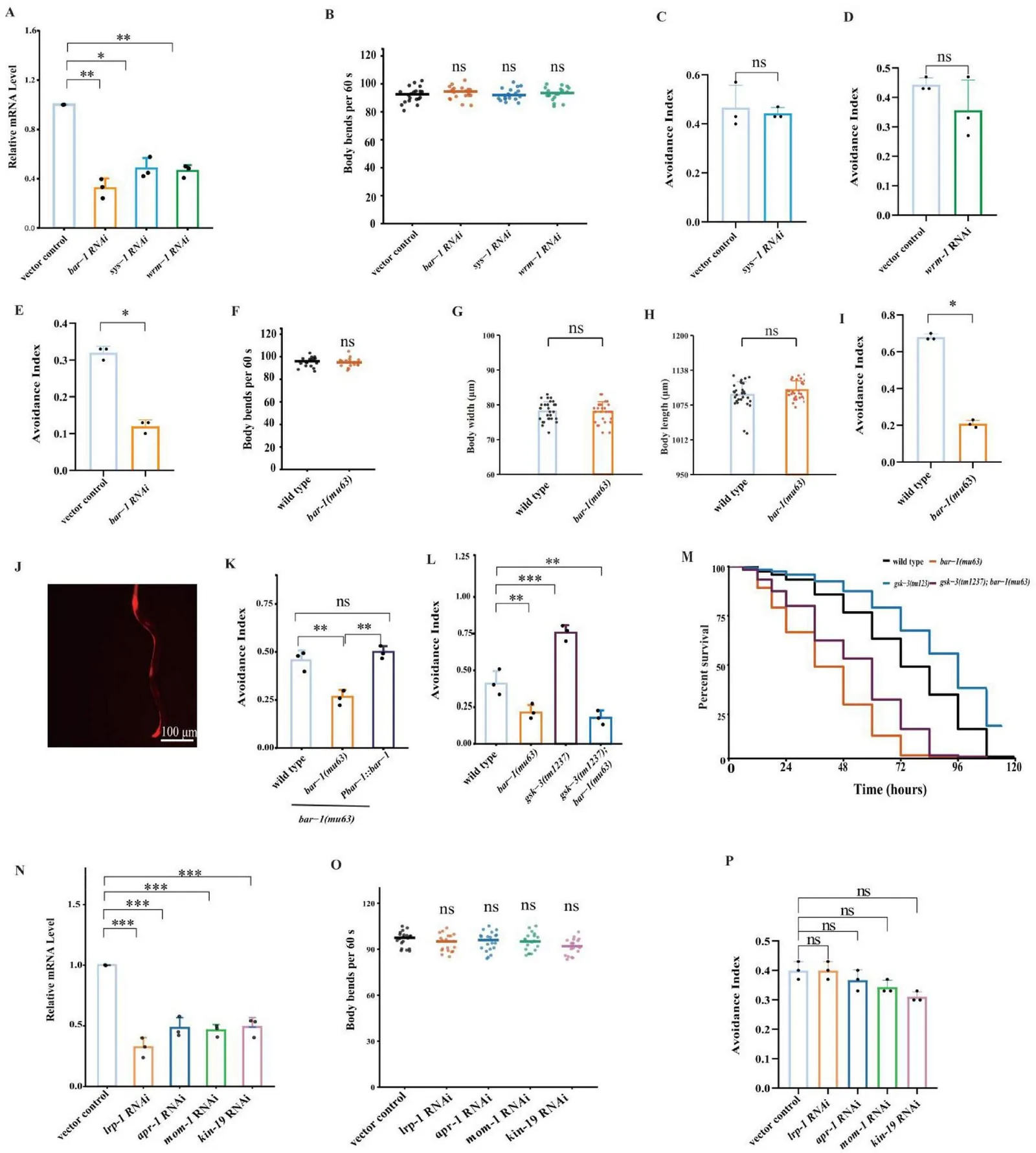
*bar-1* contributes to PA14 avoidance and functions genetically downstream of ***gsk-3*** in *Caenorhabditis elegans*. **(A)** qPCR validation of RNAi efficiency for the β-catenin homologs *bar-1*, *sys-1*, and *wrm-1*. Relative mRNA levels are shown after normalization to the vector control. **(B)** Body-bend assay of vector control, *bar-1* RNAi, *sys-1* RNAi, and *wrm-1* RNAi animals. Body bends were quantified over 60 s. **(C–E)** PA14 avoidance in vector control and animals treated with *sys-1* RNAi **(C)**, *wrm-1* RNAi **(D)**, or *bar-1* RNAi **(E)**. Avoidance behavior was quantified as the avoidance index, defined as the fraction of animals outside the PA14 lawn after 8 h. **(F)** Body-bend assay of wild-type animals and *bar-1*(mu63) animals. Body bends were quantified over 60 s. **(G)** Body width measurement of wild-type animals and *bar-1*(mu63) animals. **(H)** Body length measurement of wild-type animals and *bar-1*(mu63) animals. **(I)** PA14 avoidance in wild-type animals and *bar-1*(mu63) animals. Avoidance behavior was quantified as the avoidance index. **(J)** Representative fluorescence image of a transgenic animal carrying the co-injection marker used for identification of rescue lines. Scale bar, 100 μm. **(K)** Rescue of the avoidance defect in *bar-1*(mu63) animals by expression of wild-type *bar-1* under its endogenous promoter (*Pbar-1::bar-1*). Avoidance behavior was quantified as the avoidance index. **(L)** Epistasis analysis of *gsk-3* and *bar-1* in PA14 avoidance. Avoidance behavior was assessed in wild-type animals, *bar-1*(mu63), *gsk-3*(tm1237), and *gsk-3*(tm1237)*; bar-1*(mu63) animals. Avoidance behavior was quantified as the avoidance index. The double mutant showed an avoidance phenotype comparable to that of *bar-1*(mu63), placing *bar-1* genetically downstream of *gsk-3* in the regulation of PA14 avoidance. **(M)** Survival of wild-type animals, *bar-1*(mu63), *gsk-3*(tm1237), and *gsk-3*(tm1237)*; bar-1*(mu63) animals on PA14. This panel is presented as a complementary host-outcome phenotype and was not used as the primary basis for epistasis inference in avoidance behavior. **(N)** qPCR validation of RNAi efficiency for additional Wnt-associated genes (*lrp-1, apr-1, mom-1*, and *kin-19*). Relative mRNA levels are shown after normalization to the vector control. **(O)** Body-bend assay of vector control and animals treated with *lrp-1*, *apr-1*, *mom-1*, or *kin-19* RNAi. Body bends were quantified over 60 s. **(P)** PA14 avoidance in vector control and animals treated with *lrp-1*, *apr-1*, *mom-1*, or *kin-19* RNAi. Avoidance behavior was quantified as the avoidance index. (N–P) Presented as an extended targeted screen to assess the specificity of the core *cfz-2*–*gsk-3*–*bar-1* module identified in this study. Except for survival data, data are presented as mean ± SD. For qPCR experiments, biological replicates represented independent worm samples collected from separately prepared treatment groups. For body-bend assays, each group included three biological replicates with 30 worms per replicate. For body-size measurements, 30 animals per group were analyzed. For avoidance assays, each biological replicate represented one independent experiment performed with a distinct batch of synchronized worms; each biological replicate contained three technical replicate plates, and the mean of the technical replicates was used for statistical analysis. Statistical comparisons for avoidance assays were performed within matched experimental batches using the corresponding vector control or wild-type control included in the same experiment. Statistical significance in **(A,B,K,L,N–P)** was determined using one-way ANOVA followed by Tukey’s multiple-comparisons test. Statistical significance in **(C–I)** was determined using a two-tailed unpaired Student’s *t*-test. Survival curves in **(M)** were analyzed using the Kaplan–Meier method and compared using the log-rank test. ns, *P* ≥ 0.05; *P < 0.05; **P < 0.01; ***P < 0.001.

Next, we examined the roles of β-catenin-related genes *bar-1*, *sys-1*, and *wrm-1* in PA14 avoidance behavior. qPCR validation showed that RNAi treatment significantly reduced the relative mRNA levels of *bar-1*, *sys-1*, and *wrm-1* to 0.32 ± 0.08, 0.48 ± 0.09, and 0.46 ± 0.05, respectively, relative to the vector control, confirming effective knockdown (Figure 5A). Body-bend assays showed that worms treated with *bar-1*, *sys-1*, or *wrm-1* RNAi displayed locomotor frequencies of 94.5, 92, and 93.5 bends/min, respectively, with no significant difference from the vector control, indicating that basal locomotor ability was not affected by RNAi treatment (Figure 5B).

Avoidance assays showed that knockdown of *sys-1* (0.44 ± 0.02, *P* = 0.68; Figure 5C) or *wrm-1* (0.36 ± 0.10, *P* = 0.228; Figure 5D) did not significantly alter PA14 avoidance compared with the vector control. In contrast, among the three β-catenin homologue-related genes examined, only *bar-1* RNAi significantly reduced the avoidance index (0.12 ± 0.02 vs. 0.32 ± 0.02 in the vector control, *P* < 0.05; Figure 5E). To validate the role of *bar-1*, we further analyzed the *bar-1*(mu63) mutant. Its locomotor frequency (95 bends/min) was not significantly different from that of wild-type worms (96 bends/min), indicating normal locomotor ability (Figure 5F). In addition, *bar-1*(mu63) animals did not show significant gross body-size abnormalities compared with wild-type animals, with no significant differences in body width or body length (body width: 78.90 ± 2.72 vs. 78.10 ± 2.78 μm; body length: 1101.73 ± 16.75 vs. 1093.10 ± 23.78 μm; both *P* ≥ 0.05; Figures 5G,H). However, the *bar-1*(mu63) mutant exhibited a significantly reduced avoidance index (0.21 ± 0.02) compared with wild-type worms (0.68 ± 0.02, *P* < 0.05; Figure 5I), consistent with the RNAi phenotype.

To confirm that the reduced avoidance phenotype was attributable to loss of *bar-1*, we examined whether expression of wild-type *bar-1* under its endogenous promoter could rescue the reduced avoidance phenotype of *bar-1*(mu63) animals (Figures 5J,K). The avoidance index of the rescued line was restored to 0.50 ± 0.03, which was comparable to that of wild-type worms (0.46 ± 0.03; Figure 5K), indicating that the loss of *bar-1* underlies the avoidance defect. To determine the genetic relationship between *bar-1* and *gsk-3*, we analyzed the avoidance phenotype of the *gsk-3*(tm1237); *bar-1*(mu63) double mutant. The avoidance index of *bar-1*(mu63) animals was 0.21 ± 0.05, significantly lower than that of wild-type animals (0.53 ± 0.03, *P* < 0.01). The double mutant *gsk-3*(tm1237); *bar-1*(mu63) showed an avoidance index of 0.18 ± 0.05, which was significantly lower than that of wild-type animals but not significantly different from the *bar-1*(mu63) single mutant (Figure 5L). These results indicate that the loss of *bar-1* suppresses the high-avoidance phenotype associated with *gsk-3* mutation, placing *bar-1* genetically downstream of *gsk-3* in the regulation of avoidance behavior. This genetic relationship is consistent with the canonical Wnt/β-catenin signaling logic, in which *gsk-3* negatively regulates *bar-1* stability or activity.

To further examine whether altered activity of the Wnt/*gsk-3*/*bar-1* module was also associated with host outcome during pathogen exposure, we analyzed survival on PA14 in wild-type animals, *bar-1*(mu63) animals, *gsk-3*(tm1237) animals, and *gsk-3*(tm1237); *bar-1*(mu63) animals. Kaplan–Meier analysis showed a significant difference in survival among the four strains (Figure 5M), indicating that altered activity of these Wnt pathway components is associated with host outcome on PA14. Because survival on PA14 reflects the combined effects of behavioral avoidance and post-exposure host responses, this result was interpreted as a complementary phenotype rather than direct mechanistic evidence for avoidance regulation.

To further evaluate the contribution of Wnt-associated intracellular components, we extended the analysis to *apr-1*, *kin-19*, *mom-1*, and the functionally related gene *lrp-1*. qPCR confirmed that RNAi effectively reduced the expression of *apr-1*, *kin-19*, *mom-1*, and *lrp-1* to 0.32, 0.48, 0.46, and 0.49, respectively, relative to the vector control (Figure 5N). Body-bend assays showed that knockdown of these genes did not significantly affect basal locomotor activity, with body-bend frequencies of 93, 95, 93, and 92 bends/min for *apr-1*, *kin-19*, *mom-1*, and *lrp-1* RNAi animals, respectively, compared with 98 bends/min in the vector control (Figure 5O). Avoidance assays further showed that RNAi of *lrp-1* (0.40 ± 0.03), *apr-1* (0.37 ± 0.04), *mom-1* (0.34 ± 0.02), or *kin-19* (0.33 ± 0.04) did not significantly alter the avoidance index relative to the vector control (0.40 ± 0.03; Figure 5P). These results indicate that, under the experimental conditions tested, these additional Wnt-associated genes did not show detectable effects on PA14 avoidance. These extended screening results help define the specificity of the core *cfz-2*–*gsk-3*–*bar-1* module identified above.

### 3.6 Reporter expression patterns, exploratory network associations, and genetic hierarchy of canonical Wnt pathway genes in avoidance behavior

**FIGURE 6:**
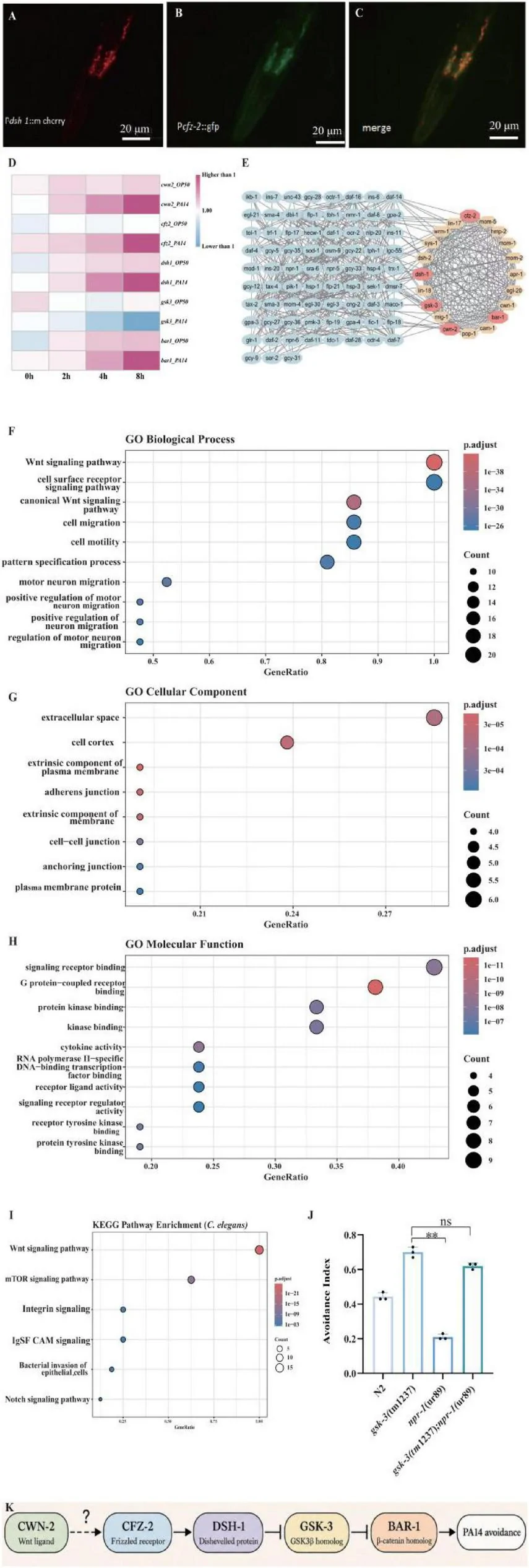
Reporter expression patterns, transcriptional responses, exploratory network associations, and genetic hierarchy of canonical Wnt signaling in pathogen avoidance. **(A–C)** Representative fluorescence images showing reporter expression in the head region. **(A)** *Pdsh-1::mCherry* fluorescence, **(B)** *Pcfz-2::GFP* fluorescence, and **(C)** merged fluorescence image. Overlapping fluorescence signals were observed in a subset of head-region cells with neuron-like morphology. Cell identity was inferred tentatively based on fluorescence morphology and anatomical position; neuron-specific marker validation was not performed. Therefore, these data indicate overlapping reporter expression patterns but do not establish neuron-specific function in PA14 avoidance. Scale bars, 20 μm. **(D)** Heatmap showing relative mRNA expression levels of selected Wnt pathway genes in wild-type animals exposed to OP50 or PA14 for 0, 2, 4, and 8 h. The analyzed genes included *cwn-2*, *cfz-2*, *dsh-1*, *gsk-3*, and *bar-1*. Relative expression levels were calculated using the 2^-ΔΔCt method, with the corresponding OP50 0 h group for each gene used as the calibrator. Values represent the mean relative expression from three independent biological replicates. Blue indicates lower relative expression, white indicates no change relative to the calibrator, and red indicates higher relative expression. **(E)** Protein–protein interaction/network association analysis generated from the predefined exploratory input gene set, integrating avoidance-associated neuronal genes, canonical Wnt pathway components, and targeted Wnt pathway genes examined in this study. Nodes represent proteins or gene products, and edges represent known or predicted functional associations. **(F–H)** Gene Ontology enrichment analysis of the exploratory input gene set used for the network analysis. Dot plots show enriched terms in Biological Process **(F)**, Cellular Component **(G)**, and Molecular Function **(H)**. Dot size indicates gene count, and color indicates adjusted significance value, shown as adjusted *P*. **(I)** KEGG pathway enrichment analysis of the exploratory input gene set used for the network analysis. Dot size indicates gene count, and color indicates adjusted significance value, shown as adjusted *P*. **(J)** Epistasis analysis of *gsk-3* and *npr-1*. Avoidance behavior was quantified as the avoidance index, defined as the fraction of animals outside the PA14 lawn after 8 h, and was compared among N2, *gsk-3*(tm1237), *npr-1*(ur89), and *gsk-3*(tm1237)*; npr-1*(ur89) animals. The double mutant showed an avoidance phenotype comparable to that of the *gsk-3* single mutant, supporting the placement of *gsk-3* downstream of, or functionally epistatic to, *npr-1* in the regulation of pathogen avoidance behavior. **(K)** Proposed working model for canonical Wnt signaling in PA14 avoidance. *cwn-2* is shown as a tentative upstream Wnt ligand because interpretation of *cwn-2* mutant phenotypes is limited by impaired locomotor activity; therefore, the *cwn-2*-related connection is indicated by a dashed line and question mark. The more strongly supported pathway involves *cfz-2* and *dsh-1*, with *dsh-1* negatively regulating *gsk-3* and *gsk-3* negatively regulating *bar-1* stability or activity, thereby contributing to PA14 avoidance. For **(J)**, data are presented as mean ± SD. Each biological replicate represented one independent experiment performed with a distinct batch of synchronized worms; each biological replicate contained three technical replicate plates, and the mean of the technical replicates was used for statistical analysis. Statistical significance was determined using one-way ANOVA followed by Tukey’s multiple-comparisons test. ns, *P* ≥ 0.05; **P < 0.01. In **(F–I)**, dot size indicates gene count and color indicates adjusted significance value, shown as adjusted *P*.

To further examine the relationship between canonical Wnt signaling and avoidance behavior, we analyzed the reporter expression patterns, network associations, and genetic hierarchy. Reporter analysis using *Pdsh-1::mCherry* and *Pcfz-2::GFP* showed that both reporters were detectable in the head region (Figures 6A,B). The merged images revealed overlapping fluorescence signals in a subset of head-region cells with neuron-like morphology (Figure 6C). Based on fluorescence morphology, cell number, and anatomical position, these cells were tentatively assigned with reference to the *C. elegans* neuronal atlas. However, because neuron-specific marker validation and cell-type-specific functional assays were not performed, these data should be interpreted as expression correlations rather than evidence for a neuron-specific site of action.

To further examine whether Wnt pathway components show dynamic transcriptional changes during bacterial exposure, we measured the expression levels of selected Wnt pathway genes in wild-type animals exposed to OP50 or PA14 for 0, 2, 4, and 8 h. Under PA14 conditions, *cwn-2*, *cfz-2*, *dsh-1*, and *bar-1* showed increased relative expression, particularly at later exposure time points, whereas *gsk-3* showed reduced relative expression, especially at 4 and 8 h (Figure 6D). In contrast, expression levels of these genes remained close to baseline under OP50 conditions. These results support that selected Wnt pathway components are transcriptionally responsive during PA14 exposure, although they do not establish neuron-specific or real-time pathway activity.

To provide an exploratory network context for the Wnt pathway components examined in this study, we performed STRING-based protein–protein interaction (PPI) analysis using a predefined input gene set. This gene set consisted of three categories: literature-curated genes associated with pathogen avoidance, sensory behavior, avoidance-related neuronal regulation, neuroimmune regulation, or stress/defense pathways; Wnt pathway genes examined in this study; and additional Wnt pathway-related genes included on the basis of published pathway annotations to provide Wnt network context. The Wnt pathway genes examined in this study included *cwn-2*, *mom-2*, *cfz-2*, mig-1, *dsh-1*, *dsh-2*, *mig-5*, *gsk-3*, *bar-1*, *sys-1*, *wrm-1*, *lrp-1*, *apr-1*, *mom-1*, and *kin-19*. Among these genes, *cwn-2*, *cfz-2*, *dsh-1*, *gsk-3*, and *bar-1* showed stronger experimental support for involvement in PA14 avoidance, whereas the other examined Wnt-associated genes were included as screened/tested components or specificity controls. The complete input gene list, selection criteria, evidence basis, and supporting references are provided in Supplementary Table 1. STRING-based PPI analysis revealed a predicted association network among the predefined input genes (Figure 6E). Because STRING networks are based on existing annotations, reported interactions, co-expression, and predicted associations, this analysis should not be interpreted as direct evidence of physical interaction or functional regulation in PA14 avoidance. Rather, the PPI network is presented as an exploratory, hypothesis-generating analysis that places the experimentally supported Wnt pathway components within a broader avoidance-, sensory-, neuroimmune-, stress- response-, and Wnt-related regulatory context.

Next, we performed GO and KEGG enrichment analyses to characterize the annotation-based functional features of the exploratory gene set used for network analysis. GO analysis showed enrichment for Wnt signaling-related terms and biological processes associated with nervous system development and neuronal migration, including motor neuron migration (Figure 6F). In the cellular component category, enriched terms included the extracellular region, plasma membrane, and cell junction (Figure 6G). In the molecular function category, enriched terms included receptor binding and G protein-coupled receptor binding (Figure 6H). KEGG analysis identified enrichment in Wnt signaling and other annotated pathways, including mTOR, integrin, and Notch signaling (Figure 6I). These enrichment results support that the analyzed gene set is annotation-linked to multiple biological processes, including neural development, cell adhesion, and intercellular interaction, and signal recognition (Baskarapantula et al., 2025; Hua et al., 2022; Li et al., 2023; Neary and Zimmermann, 2009). However, these computational analyses do not establish causal or mechanistic relationships between these pathways and PA14 avoidance behavior. Accordingly, these analyses are presented as hypothesis-generating observations to guide future experimental validation.

Because pathogen avoidance in *C. elegans* is regulated by multiple signaling pathways, including the neuropeptide receptor pathway *npr-1*, we examined the genetic relationship between Wnt signaling and *npr-1*. Epistasis analysis using the *gsk-3*(tm1237); *npr-1*(ur89) double mutant showed that its avoidance index (0.62 ± 0.02) was not significantly different from that of the *gsk-3* single mutant (0.70 ± 0.03, *P* = 0.12), but was significantly higher than that of the *npr-1* single mutant (0.21 ± 0.02, *P* < 0.01; Figure 6J). These results indicate that *gsk-3* genetically suppresses the low-avoidance phenotype of *npr-1*, supporting the placement of *gsk-3* downstream of, or functionally epistatic to, *npr-1* in the regulation of avoidance behavior.

Based on the genetic analyses described above, we propose a working model in which canonical Wnt signaling contributes to pathogen avoidance behavior in *C. elegans* (Figure 6K). In this model, *cwn-2* is shown as a tentative upstream ligand component because its interpretation is limited by locomotor defects observed in *cwn-2* mutants. The more strongly supported core pathway involves the Frizzled-family receptor *cfz-2* and the intracellular transducer *dsh-1*, with *dsh-1* negatively regulating *gsk-3*. Because *gsk-3* negatively regulates the stability or activity of the β-catenin homologue *bar-1*, inhibition of *gsk-3* is expected to relieve negative regulation of *bar-1* and allow *bar-1*-dependent signaling to promote PA14 avoidance. This genetic framework is consistent with the high-avoidance phenotype of *gsk-3*(tm1237), the reduced avoidance phenotype of *bar-1*(mu63), and the suppression of the *gsk-3*(tm1237) phenotype by *bar-1*(mu63). However, reporter signals detected in head-region cells with neuron-like morphology do not establish the precise cellular site of action, which remains to be determined.

## 4 Discussion

### 4.1 Role and regulatory mechanisms of canonical Wnt signaling in pathogen avoidance

This study provides genetic evidence that canonical Wnt signaling contributes to the regulation of avoidance behavior toward PA14 in *C. elegans*. Beyond its established roles in development and tumor biology, our findings support that canonical Wnt signaling also participates in behavioral regulation in adult animals. The combined genetic, phenotypic, and reporter expression data support the involvement of this pathway in avoidance behavior through mechanisms that are not readily explained by developmental defects alone, although developmental or physiological contributions cannot be fully excluded.

Previous studies have shown that adult *C. elegans* can modify olfactory preference after exposure to pathogenic bacteria, indicating that pathogen-related behavior is shaped, at least in part, by experience-dependent neural plasticity rather than being determined solely by developmental wiring. In this context, the canonical Wnt pathway may be associated with transcriptional changes downstream of *gsk-3* inhibition and *bar-1* stabilization. These changes may involve genes annotated for signal reception, neuronal excitability, and intercellular communication, although their direct contribution to avoidance behavior remains to be tested. Previous work showing that Wnt/Frizzled signaling regulates neuronal polarity and axon outgrowth in *C. elegans* is consistent with the possibility that Wnt pathway activity influences behavior through conserved effects on neuronal structure and organization.

Canonical Wnt signaling also interacts with other pathways associated with stress and metabolism, including p38 MAPK, TGF-β, and insulin/PI3K signaling (Barzegar Behrooz et al., 2022; Holstein, 2022). This raises the possibility that Wnt signaling may be associated with a broader regulatory framework that integrates environmental and physiological inputs during pathogen avoidance. However, the precise molecular mechanisms by which canonical Wnt signaling influences behavioral decision-making remain to be determined. Epistasis analyses are consistent with canonical Wnt/β-catenin signaling logic, supporting a model in which *dsh-1* negatively regulates *gsk-3* and *gsk-3* negatively regulates *bar-1* stability or activity. The resemblance of the *gsk-3*(tm1237); *bar-1*(mu63) double mutant to *bar-1*(mu63) further supports the placement of *bar-1* genetically downstream of *gsk-3*. The role of *cwn-2* should be interpreted with particular caution. Although *cwn-2* RNAi reduced PA14 avoidance without an obvious locomotor defect, *cwn-2*(ok895) mutants showed impaired locomotor activity, making it difficult to determine whether the altered avoidance phenotype reflects a specific defect in pathogen avoidance or a secondary consequence of compromised movement. Therefore, *cwn-2* is presented as a tentative upstream component in the working model. Future extended time-course avoidance assays will be needed to determine whether *cwn-2* mutants show delayed avoidance or fail to reach normal avoidance levels after prolonged PA14 exposure.

### 4.2 Expression patterns and possible cellular relevance

Taken together, the reporter expression and genetic data indicate that canonical Wnt signaling contributes to pathogen avoidance. The observed reporter expression pattern in a subset of head-region cells with neuron-like morphology is consistent with, but does not establish, a possible neuronal association (Mai et al., 2025; Zhang et al., 2016).

Functional enrichment analyses indicated that avoidance-related genes were annotated in categories associated with receptor binding, plasma membrane, and cell junctions, suggesting potential associations with signal reception and intercellular communication relevant to pathogen-associated cues. Enrichment in axon guidance, cell adhesion, and related neuronal terms supports that these genes are associated with annotated processes relevant to neuronal development or organization, which may be relevant to avoidance behavior. However, these enrichment results do not establish that Wnt signaling directly regulates the structure or function of avoidance circuits. This interpretation is consistent with previous studies showing that Wnt/Frizzled signaling regulates neuronal polarity and axon outgrowth in *C. elegans* (Seiradake et al., 2016; Tejeda-Muñoz and Mei, 2024); however, whether similar neuronal mechanisms contribute to PA14 avoidance behavior remains to be experimentally determined.

Epistasis analyses further placed the Wnt/*gsk-3* module genetically downstream of the *npr-1* pathway, suggesting that environmental inputs may converge on the Wnt axis through *npr-1* to influence avoidance phenotypes. Previous genetic studies showed that *npr-1*-dependent susceptibility to pathogens is largely determined by oxygen-dependent behavioral avoidance, supporting the view that the Wnt/*gsk-3* module functions downstream of a sensory-state regulator rather than as an independent immune effector pathway. Overall, canonical Wnt signaling is more cautiously interpreted as a candidate component associated with the regulation of PA14 avoidance behavior. Whether it modulates avoidance through specific neurons or connectivity-related mechanisms requires further cell-type-specific and functional validation.

### 4.3 Future directions for neuron-focused mechanistic work

Recent circuit-level research into the properties of AWB, AUA, and RMG revealed that these neurons belong to a sensorimotor pathway that regulates learned aversions to pathogens (Filipowicz et al., 2022). This provides the necessary foundation for testing how *cfz-2*, *dsh-1*, *bar-1*, and *gsk-3* function within the existing avoidance circuit. Because our current study does not directly establish the cellular site of action of *cfz-2* or *dsh-1*, neuron-specific rescue, neuron-specific RNAi, tissue-specific protein degradation, or conditional genetic approaches will be important future directions for determining whether canonical Wnt signaling functions directly within the nervous system to regulate PA14 avoidance. Subsequently, to rigorously establish the mechanisms by which canonical Wnt signaling modulates avoidance behavior at the level of neurons, cell-type-resolved causal tests should be a priority for future research. First, by using CRISPR/Cas9 to create tissue-specific knockouts or AuDA to generate auxin-inducible degrons, scientists can conditionally perturb Wnt pathway activity within a specific neuronal population to reveal its in vivo contribution to adult behavioral responses in real-time (Akay et al., 2025; Han et al., 2024). Given the genetic interactions between the Wnt and *npr-1* pathways observed above, one particularly fruitful area for future studies would be to use these techniques to perturb Wnt signaling within the RMG interneurons, which likely interact with *npr-1*, and assess the biological, behavioral, and molecular outcomes of this manipulation. Second, by employing in vivo calcium imaging or similar methods of analyzing calcium dynamics in neurons, it will be possible to assess how calcium dynamics evoked through engagement of the pathogen signal pathway are affected by Wnt signaling, thus allowing for the determination of how the molecular pathway impacting the avoidance response impacts neuronal activation via the integration of signals. Finally, by using combined perturbation of the neural circuit and quantitative behavioral analyses, researchers can clarify the role of these neurons in sensory-motor transformation and locate them within the greater network of defense behaviors, including *npr-1*, which will provide insight into how these two distinct pathways cooperate to mediate avoidance responses.

### 4.4 Limitations and outlook

This study used predominantly RNAi and conventional mutant strains, thereby hindering the ability to exclude developmental effects that may impact adult behavior due to development, such as wiring/morphology. The present genetic screen was not an exhaustive approach. First, the RNAi library screened had limited representation and limited ability for some clones to achieve efficient knockdown, leading to some “hit” genes not represented in this initial screen. Second, restricted access to specific alleles limited the ability to validate many of the possible candidates. The PA14 lawn avoidance assay is sensitive to experimental conditions, and absolute avoidance indices may vary across batches because of differences in PA14 growth state, lawn properties, plate dryness, temperature, humidity, worm synchronization, and transfer procedures (Bai et al., 2021; Marquina-Solis et al., 2024). Therefore, values from different figure panels should not be directly compared. In this study, behavioral comparisons were performed within matched experimental batches containing the corresponding wild-type or vector control, and conclusions were based on reproducible directional changes across independent experiments and consistency among RNAi, mutant, rescue, and epistasis analyses. Although the absolute avoidance index of *gsk-3*(tm1237) varied between batches, RNAi, mutant, and epistasis analyses consistently supported a negative regulatory role for *gsk-3* in the Wnt pathway. Nevertheless, this behavioral variability remains a limitation of the assay and should be considered when interpreting absolute avoidance indices across independent experiments.

Future studies should improve their reproducibility through the use of randomized block designs, double-blinded scoring, and cross-paradigm validation. Finally, the lack of synchronized measurements of “molecule–cell–circuit–behavior” within the same individual reduces the strength of causal inference across levels. The NPR-1-mediated avoidance of pathogens can be inhibited by mucoid bacteria and bacteria that secrete exopolysaccharides, which renders it important to determine whether the Wnt-dependent phenotype is maintained regardless of the bacterial status that changes the physical characteristics of the lawn (Reddy et al., 2009). More generally, NPR-1-expressing neurons appear to regulate the innate immune response; therefore, avoidance behavior and innate defense should be considered as integrated parts of a collective neuroimmune program.

At the level of function/gene, transcriptomic and chromatin accessibility profiling resolved by cell type and through the use of *bar-1*/TCF ChIP-seq will be necessary to identify direct targets for gene networks specifically in relation to avoidance behaviors. At the circuit level, imaging combined with optical manipulation, as well as functional imaging, will help determine how Wnt signaling affects the weighting and temporal dependency of nodes such as AVA/AIY/AIZ. At the pathway level, tests of interactions between Wnt and the p38, TGF-β, and insulin/PI3K signaling pathways will allow for the determination of whether Wnt and these pathways are integrated in parallel or amplify serially. At the level of exposure, expanding avoidance assessment to multiple pathogens (e.g., PA14, S. marcescens) as well as inorganic toxic substances (e.g., arsenic and cadmium) will allow for the assessment of the generality versus contextual specificity of the Wnt–*npr-1* framework. From an evolutionary standpoint, conducting multigenerational selection experiments will allow for the testing of whether long-term pathogen exposure leads to genetic assimilation or epigenetic memory that is reversible. Lastly, studies conducted using other species and higher organisms may assist in determining whether Wnt signaling regulation of synaptic plasticity and avoidance of threats is evolutionarily conserved across olfactory-evoking circuits. Collectively, these approaches may help test whether the current genetic and computational associations reflect causal mechanisms at a time- and cell-type-resolved level, thereby providing a more rigorous framework for evaluating possible interactions among neural, immune, metabolic, and behavioral processes.

## 5 Conclusion

In conclusion, our study provides genetic evidence that canonical Wnt signaling contributes to the regulation of avoidance behavior toward *Pseudomonas aeruginosa* PA14 in *C. elegans*. Genetic analyses implicated *cfz-2*, *dsh-1*, and *bar-1* in this behavior, whereas *gsk-3* acted as a negative regulator. RNAi results indicated a possible involvement of *cwn-2*, but its role should be interpreted cautiously because *cwn-2* mutant phenotypes were confounded by impaired locomotor activity. Epistasis analyses further supported a model in which *cfz-2* functions genetically upstream of *gsk-3* and *bar-1* in the regulation of avoidance behavior. Reporter analyses showed overlapping expression of *cfz-2* and *dsh-1* in a subset of head-region cells with neuron-like morphology, which is consistent with a possible but unproven neuronal association. Systems-level analyses showed annotation-based associations between Wnt pathway genes and avoidance-related neuronal or regulatory gene sets, providing exploratory hypotheses rather than direct mechanistic evidence. Genetic interaction analyses further placed the Wnt/*gsk-3*/*bar-1* module downstream of the central neuropeptide receptor pathway *npr-1*, supporting that canonical Wnt signaling functions within a broader behavioral regulatory network rather than as an isolated immune effector pathway. These findings support a role for canonical Wnt signaling in PA14 avoidance. However, the current data do not establish a neuron-specific site of action or a circuit-level mechanism, and should therefore be interpreted as genetic association and expression correlation rather than direct evidence for a neuronal mechanism. Further cell-type-specific rescue, knockdown, or conditional manipulation experiments will be required to determine the precise cellular site and mechanism of action.

## Data Availability

The original contributions presented in the study are included in the article/Supplementary material, further inquiries can be directed to the corresponding authors.
