# Supplementary material for: Regulation of Caenorhabditis elegans avoidance behavior toward Pseudomonas aeruginosa PA14 by the canonical Wnt signaling pathway
Source: Front Mol Neurosci. 2026 Aug 11;19:1851614. doi: 10.3389/fnmol.2026.1851614 (PMC13503345; doi:10.3389/fnmol.2026.1851614)
Supplement: Supplementary file 1 [file Table_1.docx]

**Supplementary Table S1.Predefined input gene set used for STRING-based PPI analysis**

| Gene | Selection criterion / evidence basis | Supporting reference(s) to verify | Included in this study |
| --- | --- | --- | --- |
| *ikb-1* | Selected because published literature reported association with pathogen avoidance, sensory behavior, avoidance-related neuronal regulation, neuroimmune regulation, or stress/defense pathways. | Pujol et al., Current Biology, 2001. “A reverse genetic analysis of components of the Toll signaling pathway in *C. elegans*.” PMID: 11516642 | No; background gene for network context |
| *ins-7* | Selected because published literature reported association with pathogen avoidance, sensory behavior, avoidance-related neuronal regulation, neuroimmune regulation, or stress/defense pathways. | Chen et al., Neuron, 2013. “Two Insulin-like Peptides Antagonistically Regulate Aversive Olfactory Learning in *C. elegans.*” DOI: 10.1016/j.neuron.2012.11.025 | No; background gene for network context |
| *unc-43* | Selected because published literature reported association with pathogen avoidance, sensory behavior, avoidance-related neuronal regulation, neuroimmune regulation, or stress/defense pathways. | Qin et al., Journal of Neuroscience, 2013. “A Neuronal Signaling Pathway of CaMKII and Gqα Regulates Experience-Dependent Transcription of tph-1.” DOI: 10.1523/JNEUROSCI.2355-12.2013 | No; background gene for network context |
| *gcy-28* | Selected because published literature reported association with pathogen avoidance, sensory behavior, avoidance-related neuronal regulation, neuroimmune regulation, or stress/defense pathways. | Shinkai et al., Journal of Neuroscience, 2011. “Behavioral Choice between Conflicting Alternatives Is Regulated by a Receptor Guanylyl Cyclase, GCY-28, and a Receptor Tyrosine Kinase, SCD-2, in AIA Interneurons of *Caenorhabditis elegans*.” DOI: 10.1523/JNEUROSCI.4691-10.2011 | No; background gene for network context |
| *octr-1* | Selected because published literature reported association with pathogen avoidance, sensory behavior, avoidance-related neuronal regulation, neuroimmune regulation, or stress/defense pathways. | Cao et al., Cell Reports, 2017. “Distinct Roles of Sensory Neurons in Mediating Pathogen Avoidance and Neuropeptide-Dependent Immune Regulation.” DOI: 10.1016/j.celrep.2017.10.050 | No; background gene for network context |
| *daf-16* | Selected because published literature reported association with pathogen avoidance, sensory behavior, avoidance-related neuronal regulation, neuroimmune regulation, or stress/defense pathways. | Meisel et al., Cell, 2014. “Chemosensation of bacterial secondary metabolites modulates neuroendocrine signaling and behavior of *C. elegans.*” PMID: 25303524 | No; background gene for network context |
| *ins-6* | Selected because published literature reported association with pathogen avoidance, sensory behavior, avoidance-related neuronal regulation, neuroimmune regulation, or stress/defense pathways. | Chen et al., Neuron, 2013. “Two Insulin-like Peptides Antagonistically Regulate Aversive Olfactory Learning in *C. elegans.”* DOI: 10.1016/j.neuron.2012.11.025 | No; background gene for network context |
| *daf-14* | Selected because published literature reported association with pathogen avoidance, sensory behavior, avoidance-related neuronal regulation, neuroimmune regulation, or stress/defense pathways. | Meisel et al., Cell, 2014. “Chemosensation of Bacterial Secondary Metabolites Modulates Neuroendocrine Signaling and Behavior of *C. elegans*.” DOI: 10.1016/j.cell.2014.09.011 | No; background gene for network context |
| *egl-21* | Selected because published literature reported association with pathogen avoidance, sensory behavior, avoidance-related neuronal regulation, neuroimmune regulation, or stress/defense pathways. | Marquina-Solis et al., Cell Reports, 2024. “Antagonism between neuropeptides and monoamines in a distributed circuit for pathogen avoidance.” DOI: 10.1016/j.celrep.2024.114042 | No; background gene for network context |
| *sma-4* | Selected because published literature reported association with pathogen avoidance, sensory behavior, avoidance-related neuronal regulation, neuroimmune regulation, or stress/defense pathways. | Madhu et al., eLife, 2023. “The DBL-1/TGF-β signaling pathway tailors behavioral and molecular host responses to a variety of bacteria in *Caenorhabditis elegans*.” DOI: 10.7554/eLife.75831 | No; background gene for network context |
| *dbl-1* | Selected because published literature reported association with pathogen avoidance, sensory behavior, avoidance-related neuronal regulation, neuroimmune regulation, or stress/defense pathways. | Madhu et al., eLife, 2023. “The DBL-1/TGF-β signaling pathway tailors behavioral and molecular host responses to a variety of bacteria in *Caenorhabditis elegans*.” DOI: 10.7554/eLife.75831 | No; background gene for network context |
| *flp-1* | Selected because published literature reported association with pathogen avoidance, sensory behavior, avoidance-related neuronal regulation, neuroimmune regulation, or stress/defense pathways. | Marquina-Solis et al., Cell Reports, 2024. “Antagonism between neuropeptides and monoamines in a distributed circuit for pathogen avoidance.” DOI: 10.1016/j.celrep.2024.114042 | No; background gene for network context |
| *tbh-1* | Selected because published literature reported association with pathogen avoidance, sensory behavior, avoidance-related neuronal regulation, neuroimmune regulation, or stress/defense pathways. | Marquina-Solis et al., Cell Reports, 2024. “Antagonism between neuropeptides and monoamines in a distributed circuit for pathogen avoidance.” DOI: 10.1016/j.celrep.2024.114042 | No; background gene for network context |
| *nmr-1* | Selected because published literature reported association with pathogen avoidance, sensory behavior, avoidance-related neuronal regulation, neuroimmune regulation, or stress/defense pathways. | Ha et al., Neuron, 2010. “Functional organization of a neural network for aversive olfactory learning in *C. elegans*.” PMID: 21172617 | No; background gene for network context |
| *daf-8* | Selected because published literature reported association with pathogen avoidance, sensory behavior, avoidance-related neuronal regulation, neuroimmune regulation, or stress/defense pathways. | Meisel et al., Cell, 2014. “Chemosensation of bacterial secondary metabolites modulates neuroendocrine signaling and behavior of *C. elegans*.” PMID: 25303524 | No; background gene for network context |
| *gpa-2* | Selected because published literature reported association with pathogen avoidance, sensory behavior, avoidance-related neuronal regulation, neuroimmune regulation, or stress/defense pathways. | Meisel et al., Cell, 2014. “Chemosensation of bacterial secondary metabolites modulates neuroendocrine signaling and behavior of *C. elegans*.” PMID: 25303524 | No; background gene for network context |
| *tol-1* | Selected because published literature reported association with pathogen avoidance, sensory behavior, avoidance-related neuronal regulation, neuroimmune regulation, or stress/defense pathways. | Pujol et al., Current Biology, 2001. “A reverse genetic analysis of components of the Toll signaling pathway in *C. elegans*.” PMID: 11516642 | No; background gene for network context |
| *trf-1* | Selected because published literature reported association with pathogen avoidance, sensory behavior, avoidance-related neuronal regulation, neuroimmune regulation, or stress/defense pathways. | Pujol et al., Current Biology, 2001. “A reverse genetic analysis of components of the Toll signaling pathway in *C. elegans*.” PMID: 11516642 | No; background gene for network context |
| *flp-17* | Selected because published literature reported association with pathogen avoidance, sensory behavior, avoidance-related neuronal regulation, neuroimmune regulation, or stress/defense pathways. | Hussey et al., PLoS Genetics, 2018. “Oxygen-sensing neurons reciprocally regulate peripheral lipid metabolism via neuropeptide signaling in *Caenorhabditis elegans.”* DOI: 10.1371/journal.pgen.1007305 | No; background gene for network context |
| *hecw-1* | Selected because published literature reported association with pathogen avoidance, sensory behavior, avoidance-related neuronal regulation, neuroimmune regulation, or stress/defense pathways. | Chang et al., Nature, 2011. “Natural Polymorphisms in *C. elegans* HECW-1 E3 Ligase Affect Pathogen Avoidance Behaviour.” DOI: 10.1038/nature10643 | No; background gene for network context |
| *daf-1* | Selected because published literature reported association with pathogen avoidance, sensory behavior, avoidance-related neuronal regulation, neuroimmune regulation, or stress/defense pathways. | Meisel et al., Cell, 2014. “Chemosensation of bacterial secondary metabolites modulates neuroendocrine signaling and behavior of *C. elegans*.” PMID: 25303524 | No; background gene for network context |
| *ocr-2* | Selected because published literature reported association with pathogen avoidance, sensory behavior, avoidance-related neuronal regulation, neuroimmune regulation, or stress/defense pathways. | Reddy et al., Science, 2009. “A Polymorphism in npr-1 Is a Behavioral Determinant of Pathogen Susceptibility in *C. elegans*.” DOI: 10.1126/science.1166527 | No; background gene for network context |
| *nlp-20* | Selected because published literature reported association with pathogen avoidance, sensory behavior, avoidance-related neuronal regulation, neuroimmune regulation, or stress/defense pathways. | Cao et al., Cell Reports, 2017. “Distinct Roles of Sensory Neurons in Mediating Pathogen Avoidance and Neuropeptide-Dependent Immune Regulation.” DOI: 10.1016/j.celrep.2017.10.050 | No; background gene for network context |
| *ins-11* | Selected because published literature reported association with pathogen avoidance, sensory behavior, avoidance-related neuronal regulation, neuroimmune regulation, or stress/defense pathways. | Lee and Mylonakis, Cell Reports, 2017. “An Intestine-Derived Neuropeptide Controls Avoidance Behavior in *Caenorhabditis elegans*.” DOI: 10.1016/j.celrep.2017.08.053 | No; background gene for network context |
| *daf-4* | Selected because published literature reported association with pathogen avoidance, sensory behavior, avoidance-related neuronal regulation, neuroimmune regulation, or stress/defense pathways. | Meisel et al., Cell, 2014. “Chemosensation of bacterial secondary metabolites modulates neuroendocrine signaling and behavior of *C. elegans*.” PMID: 25303524 | No; background gene for network context |
| *gcy-5* | Selected because published literature reported association with pathogen avoidance, sensory behavior, avoidance-related neuronal regulation, neuroimmune regulation, or stress/defense pathways. | Ortiz et al., Current Biology, 2009. “Lateralized Gustatory Behavior of *C. elegans* Is Controlled by Specific Receptor-Type Guanylyl Cyclases.” DOI: 10.1016/j.cub.2009.05.043 | No; background gene for network context |
| *gcy-35* | Selected because published literature reported association with pathogen avoidance, sensory behavior, avoidance-related neuronal regulation, neuroimmune regulation, or stress/defense pathways. | Reddy et al., Science, 2009. “A Polymorphism in npr-1 Is a Behavioral Determinant of Pathogen Susceptibility in *C. elegans*.” DOI: 10.1126/science.1166527 | No; background gene for network context |
| *sod-1* | Selected because published literature reported association with pathogen avoidance, sensory behavior, avoidance-related neuronal regulation, neuroimmune regulation, or stress/defense pathways. | Yu et al., iScience, 2022. “Glutamate signaling mediates *C. elegans* behavioral plasticity to Pseudomonas.” PMID: 35252815 | No; background gene for network context |
| *osm-9* | Selected because published literature reported association with pathogen avoidance, sensory behavior, avoidance-related neuronal regulation, neuroimmune regulation, or stress/defense pathways. | Reddy et al., Science, 2009. “A Polymorphism in npr-1 Is a Behavioral Determinant of Pathogen Susceptibility in *C. elegans*.” DOI: 10.1126/science.1166527 | No; background gene for network context |
| *gcy-22* | Selected because published literature reported association with pathogen avoidance, sensory behavior, avoidance-related neuronal regulation, neuroimmune regulation, or stress/defense pathways. | Ortiz et al., Current Biology, 2009. “Lateralized Gustatory Behavior of *C. elegans* Is Controlled by Specific Receptor-Type Guanylyl Cyclases.” DOI: 10.1016/j.cub.2009.05.043 | No; background gene for network context |
| *tph-1* | Selected because published literature reported association with pathogen avoidance, sensory behavior, avoidance-related neuronal regulation, neuroimmune regulation, or stress/defense pathways. | Zhang et al., Nature, 2005. “Pathogenic bacteria induce aversive olfactory learning in *C. elegans*.” PMID: 16281027 | No; background gene for network context |
| *lgc-55* | Selected because published literature reported association with pathogen avoidance, sensory behavior, avoidance-related neuronal regulation, neuroimmune regulation, or stress/defense pathways. | Pirri et al., Neuron, 2009. “A Tyramine-Gated Chloride Channel Coordinates Distinct Motor Programs of a *Caenorhabditis elegans* Escape Response.” DOI: 10.1016/j.neuron.2009.04.029 | No; background gene for network context |
| *mod-1* | Selected because published literature reported association with pathogen avoidance, sensory behavior, avoidance-related neuronal regulation, neuroimmune regulation, or stress/defense pathways. | Ha et al., Neuron, 2010. “Functional organization of a neural network for aversive olfactory learning in *C. elegans*.” PMID: 21172617 | No; background gene for network context |
| *ins-20* | Selected because published literature reported association with pathogen avoidance, sensory behavior, avoidance-related neuronal regulation, neuroimmune regulation, or stress/defense pathways. | Pu et al., Nature Communications, 2023. “Dissecting the genetic landscape of GPCR signaling through phenotypic profiling in *C. elegans*.” DOI: 10.1038/s41467-023-44177-z | No; background gene for network context |
| *npr-1* | Selected because published literature reported association with pathogen avoidance, sensory behavior, avoidance-related neuronal regulation, neuroimmune regulation, or stress/defense pathways. | Reddy et al., Science, 2009. “A Polymorphism in npr-1 Is a Behavioral Determinant of Pathogen Susceptibility in *C. elegans*.” DOI: 10.1126/science.1166527 | No; background gene for network context |
| *sra-6* | Selected because published literature reported association with pathogen avoidance, sensory behavior, avoidance-related neuronal regulation, neuroimmune regulation, or stress/defense pathways. | Hilliard et al., Current Biology, 2004. “In vivo imaging of *C. elegans* ASH neurons: cellular response and adaptation to chemical repellents.” DOI: 10.1016/j.cub.2004.10.041 | No; background gene for network context |
| *npr-5* | Selected because published literature reported association with pathogen avoidance, sensory behavior, avoidance-related neuronal regulation, neuroimmune regulation, or stress/defense pathways. | Marquina-Solis et al., Cell Reports, 2024. “Antagonism between neuropeptides and monoamines in a distributed circuit for pathogen avoidance.” DOI: 10.1016/j.celrep.2024.114042 | No; background gene for network context |
| *gcy-33* | Selected because published literature reported association with pathogen avoidance, sensory behavior, avoidance-related neuronal regulation, neuroimmune regulation, or stress/defense pathways. | Brandt and Ringstad, Current Biology, 2015. “Toll-like Receptor Signaling Promotes Development and Function of Sensory Neurons Required for a *C. elegans* Pathogen-Avoidance Behavior.” DOI: 10.1016/j.cub.2015.07.037 | No; background gene for network context |
| *hsp-4* | Selected because published literature reported association with pathogen avoidance, sensory behavior, avoidance-related neuronal regulation, neuroimmune regulation, or stress/defense pathways. | Hernandez-Lima et al., Frontiers in Molecular Neuroscience, 2022. “The AMPylase FIC-1 modulates TGF-β signaling in *Caenorhabditis elegans*.” DOI: 10.3389/fnmol.2022.912734 | No; background gene for network context |
| *trx-1* | Selected because published literature reported association with pathogen avoidance, sensory behavior, avoidance-related neuronal regulation, neuroimmune regulation, or stress/defense pathways. | Hao et al., eLife, 2018. “Thioredoxin shapes the *C. elegans* sensory response to Pseudomonas-produced nitric oxide.” PMID: 30014846 | No; background gene for network context |
| *gcy-12* | Selected because published literature reported association with pathogen avoidance, sensory behavior, avoidance-related neuronal regulation, neuroimmune regulation, or stress/defense pathways. | Park et al., PLoS Genetics, 2020. “Immediate activation of chemosensory neuron gene expression by bacterial metabolites is selectively induced by distinct cyclic GMP-dependent pathways in *Caenorhabditis elegans*” PMID: 32776934 | No; background gene for network context |
| *tax-4* | Selected because published literature reported association with pathogen avoidance, sensory behavior, avoidance-related neuronal regulation, neuroimmune regulation, or stress/defense pathways. | Meisel et al., Cell, 2014. “Chemosensation of bacterial secondary metabolites modulates neuroendocrine signaling and behavior of *C. elegans.*” PMID: 25303524 | No; background gene for network context |
| *pik-1* | Selected because published literature reported association with pathogen avoidance, sensory behavior, avoidance-related neuronal regulation, neuroimmune regulation, or stress/defense pathways. | Pujol et al., Current Biology, 2001. “A reverse genetic analysis of components of the Toll signaling pathway in C. elegans.” PMID: 11516642 | No; background gene for network context |
| *hsp-1* | Selected because published literature reported association with pathogen avoidance, sensory behavior, avoidance-related neuronal regulation, neuroimmune regulation, or stress/defense pathways. | Hernandez-Lima et al., Frontiers in Molecular Neuroscience, 2022. “The AMPylase FIC-1 modulates TGF-β signaling in *Caenorhabditis elegans*.” DOI: 10.3389/fnmol.2022.912734 | No; background gene for network context |
| *flp-21* | Selected because published literature reported association with pathogen avoidance, sensory behavior, avoidance-related neuronal regulation, neuroimmune regulation, or stress/defense pathways. | Singh and Aballay, Developmental Cell, 2019. “Microbial Colonization Activates an Immune Fight-and-Flight Response via Neuroendocrine Signaling.” DOI: 10.1016/j.devcel.2019.02.001 | No; background gene for network context |
| *hsp-3* | Selected because published literature reported association with pathogen avoidance, sensory behavior, avoidance-related neuronal regulation, neuroimmune regulation, or stress/defense pathways. | Shen et al., Cell, 2001. “Complementary Signaling Pathways Regulate the Unfolded Protein Response and Are Required for *C. elegans* Development.” DOI: 10.1016/S0092-8674(01)00612-2 | No; background gene for network context |
| *sek-1* | Selected because published literature reported association with pathogen avoidance, sensory behavior, avoidance-related neuronal regulation, neuroimmune regulation, or stress/defense pathways. | Shivers et al., Cell Host & Microbe, 2009. “Tissue-specific activities of an immune signaling module regulate physiological responses to pathogenic and nutritional bacteria in *C. elegans*.” DOI: 10.1016/j.chom.2009.09.001 | No; background gene for network context |
| *dmsr-7* | Selected because published literature reported association with pathogen avoidance, sensory behavior, avoidance-related neuronal regulation, neuroimmune regulation, or stress/defense pathways. | Marquina-Solis et al., Cell Reports, 2024. “Antagonism between neuropeptides and monoamines in a distributed circuit for pathogen avoidance.” DOI: 10.1016/j.celrep.2024.114042 | No; background gene for network context |
| *tax-2* | Selected because published literature reported association with pathogen avoidance, sensory behavior, avoidance-related neuronal regulation, neuroimmune regulation, or stress/defense pathways. | Meisel et al., Cell, 2014. “Chemosensation of bacterial secondary metabolites modulates neuroendocrine signaling and behavior of *C. elegans*.” PMID: 25303524 | No; background gene for network context |
| *sma-3* | Selected because published literature reported association with pathogen avoidance, sensory behavior, avoidance-related neuronal regulation, neuroimmune regulation, or stress/defense pathways. | Madhu et al., eLife, 2023. “The DBL-1/TGF-β signaling pathway tailors behavioral and molecular host responses to a variety of bacteria in *Caenorhabditis elegans*.” DOI: 10.7554/eLife.75831 | No; background gene for network context |
| *mom-4* | Selected because published literature reported association with pathogen avoidance, sensory behavior, avoidance-related neuronal regulation, neuroimmune regulation, or stress/defense pathways. | Xu et al., Protein & Cell, 2013. “Caenorhabditis elegans mom-4 is required for the activation of the p38 MAPK signaling pathway in the response to Pseudomonas aeruginosa infection.” DOI: 10.1007/s13238-012-2080-z | No; background gene for network context |
| *egl-30* | Selected because published literature reported association with pathogen avoidance, sensory behavior, avoidance-related neuronal regulation, neuroimmune regulation, or stress/defense pathways. | Qin et al., Journal of Neuroscience, 2013. “A Neuronal Signaling Pathway of CaMKII and Gqα Regulates Experience-Dependent Transcription of tph-1.” DOI: 10.1523/JNEUROSCI.2355-12.2013 | No; background gene for network context |
| *egl-3* | Selected because published literature reported association with pathogen avoidance, sensory behavior, avoidance-related neuronal regulation, neuroimmune regulation, or stress/defense pathways. | Marquina-Solis et al., Cell Reports, 2024. “Antagonism between neuropeptides and monoamines in a distributed circuit for pathogen avoidance.” DOI: 10.1016/j.celrep.2024.114042 | No; background gene for network context |
| *cng-2* | Selected because published literature reported association with pathogen avoidance, sensory behavior, avoidance-related neuronal regulation, neuroimmune regulation, or stress/defense pathways. | Park et al., PLoS Genetics, 2020. “Immediate activation of chemosensory neuron gene expression by bacterial metabolites is selectively induced by distinct cyclic GMP-dependent pathways in *Caenorhabditis elegans*” PMID: 32776934 | No; background gene for network context |
| *daf-3* | Selected because published literature reported association with pathogen avoidance, sensory behavior, avoidance-related neuronal regulation, neuroimmune regulation, or stress/defense pathways. | Meisel et al., Cell, 2014. “Chemosensation of bacterial secondary metabolites modulates neuroendocrine signaling and behavior of *C. elegans*.” PMID: 25303524 | No; background gene for network context |
| *maco-1* | Selected because published literature reported association with pathogen avoidance, sensory behavior, avoidance-related neuronal regulation, neuroimmune regulation, or stress/defense pathways. | Kaletsky et al., Nature, 2020. *“C. elegans* interprets bacterial non-coding RNAs to learn pathogenic avoidance.” DOI: 10.1038/s41586-020-2699-5 | No; background gene for network context |
| *gpa-3* | Selected because published literature reported association with pathogen avoidance, sensory behavior, avoidance-related neuronal regulation, neuroimmune regulation, or stress/defense pathways. | Meisel et al., Cell, 2014. “Chemosensation of bacterial secondary metabolites modulates neuroendocrine signaling and behavior of *C. elegans*.” PMID: 25303524 | No; background gene for network context |
| *gcy-27* | Selected because published literature reported association with pathogen avoidance, sensory behavior, avoidance-related neuronal regulation, neuroimmune regulation, or stress/defense pathways. | Hao et al., eLife, 2018. “Thioredoxin shapes the *C. elegans* sensory response to Pseudomonas-produced nitric oxide.” PMID: 30014846 | No; background gene for network context |
| *gcy-36* | Selected because published literature reported association with pathogen avoidance, sensory behavior, avoidance-related neuronal regulation, neuroimmune regulation, or stress/defense pathways. | Gray et al., Nature, 2004. “Oxygen Sensation and Social Feeding Mediated by a *C. elegans* Guanylate Cyclase Homologue.” DOI: 10.1038/nature02714 | No; background gene for network context |
| *pmk-3* | Selected because published literature reported association with pathogen avoidance, sensory behavior, avoidance-related neuronal regulation, neuroimmune regulation, or stress/defense pathways. | Huang et al., G3, 2023. “Multiple p38/JNK mitogen-activated protein kinase pathways regulate salt chemotaxis learning in *Caenorhabditis elegans*.” DOI: 10.1093/g3journal/jkad129 | No; background gene for network context |
| *flp-19* | Selected because published literature reported association with pathogen avoidance, sensory behavior, avoidance-related neuronal regulation, neuroimmune regulation, or stress/defense pathways. | Pu et al., Nature Communications, 2023. “Dissecting the genetic landscape of GPCR signaling through phenotypic profiling in *C. elegans*.” DOI: 10.1038/s41467-023-44177-z | No; background gene for network context |
| *gpa-4* | Selected because published literature reported association with pathogen avoidance, sensory behavior, avoidance-related neuronal regulation, neuroimmune regulation, or stress/defense pathways. | Osuna-Luque et al., MicroPublication Biology, 2021. “Widespread gpa-4 promoter-driven expression during *Caenorhabditis elegans* development.” DOI: 10.17912/micropub.biology.000440 | No; background gene for network context |
| *fic-1* | Selected because published literature reported association with pathogen avoidance, sensory behavior, avoidance-related neuronal regulation, neuroimmune regulation, or stress/defense pathways. | Hernandez-Lima et al., Frontiers in Molecular Neuroscience, 2022. “The AMPylase FIC-1 modulates TGF-β signaling in *Caenorhabditis elegans*.” DOI: 10.3389/fnmol.2022.912734 | No; background gene for network context |
| *flp-18* | Selected because published literature reported association with pathogen avoidance, sensory behavior, avoidance-related neuronal regulation, neuroimmune regulation, or stress/defense pathways. | Singh and Aballay, Developmental Cell, 2019. “Microbial Colonization Activates an Immune Fight-and-Flight Response via Neuroendocrine Signaling.” DOI: 10.1016/j.devcel.2019.02.001 | No; background gene for network context |
| *glr-1* | Selected because published literature reported association with pathogen avoidance, sensory behavior, avoidance-related neuronal regulation, neuroimmune regulation, or stress/defense pathways. | Yu et al., iScience, 2022. “Glutamate signaling mediates *C. elegans* behavioral plasticity to Pseudomonas.” PMID: 35252815 | No; background gene for network context |
| *daf-2* | Selected because published literature reported association with pathogen avoidance, sensory behavior, avoidance-related neuronal regulation, neuroimmune regulation, or stress/defense pathways. | Chen et al., Neuron, 2013. “Two Insulin-like Peptides Antagonistically Regulate Aversive Olfactory Learning in *C. elegans*.” DOI: 10.1016/j.neuron.2012.11.025 | No; background gene for network context |
| *npr-6* | Selected because published literature reported association with pathogen avoidance, sensory behavior, avoidance-related neuronal regulation, neuroimmune regulation, or stress/defense pathways. | Marquina-Solis et al., Cell Reports, 2024. “Antagonism between neuropeptides and monoamines in a distributed circuit for pathogen avoidance.” DOI: 10.1016/j.celrep.2024.114042 | No; background gene for network context |
| *daf-11* | Selected because published literature reported association with pathogen avoidance, sensory behavior, avoidance-related neuronal regulation, neuroimmune regulation, or stress/defense pathways. | Meisel et al., Cell, 2014. “Chemosensation of bacterial secondary metabolites modulates neuroendocrine signaling and behavior of *C. elegans*.” PMID: 25303524 | No; background gene for network context |
| *tdc-1* | Selected because published literature reported association with pathogen avoidance, sensory behavior, avoidance-related neuronal regulation, neuroimmune regulation, or stress/defense pathways. | Marquina-Solis et al., Cell Reports, 2024. “Antagonism between neuropeptides and monoamines in a distributed circuit for pathogen avoidance.” DOI: 10.1016/j.celrep.2024.114042 | No; background gene for network context |
| *daf-28* | Selected because published literature reported association with pathogen avoidance, sensory behavior, avoidance-related neuronal regulation, neuroimmune regulation, or stress/defense pathways. | Pu et al., Nature Communications, 2023. “Dissecting the genetic landscape of GPCR signaling through phenotypic profiling in *C. elegans*.” DOI: 10.1038/s41467-023-44177-z | No; background gene for network context |
| *odr-4* | Selected because published literature reported association with pathogen avoidance, sensory behavior, avoidance-related neuronal regulation, neuroimmune regulation, or stress/defense pathways. | Dwyer et al., Cell, 1998. “Odorant Receptor Localization to Olfactory Cilia Is Mediated by ODR-4, a Novel Membrane-Associated Protein.” DOI: 10.1016/S0092-8674(00)81173-3 | No; background gene for network context |
| *daf-7* | Selected because published literature reported association with pathogen avoidance, sensory behavior, avoidance-related neuronal regulation, neuroimmune regulation, or stress/defense pathways. | Park et al., PLoS Genetics, 2020. “Immediate activation of chemosensory neuron gene expression by bacterial metabolites is selectively induced by distinct cyclic GMP-dependent pathways in *Caenorhabditis elegans*” PMID: 32776934 | No; background gene for network context |
| *gcy-9* | Selected because published literature reported association with pathogen avoidance, sensory behavior, avoidance-related neuronal regulation, neuroimmune regulation, or stress/defense pathways. | Brandt and Ringstad, Current Biology, 2015. “Toll-like Receptor Signaling Promotes Development and Function of Sensory Neurons Required for a *C. elegans* Pathogen-Avoidance Behavior.” DOI: 10.1016/j.cub.2015.07.037 | No; background gene for network context |
| *ser-2* | Selected because published literature reported association with pathogen avoidance, sensory behavior, avoidance-related neuronal regulation, neuroimmune regulation, or stress/defense pathways. | Wragg et al., Journal of Neuroscience, 2007. “Tyramine and Octopamine Independently Inhibit Serotonin-Stimulated Aversive Behaviors in *Caenorhabditis elegans*.” DOI: 10.1523/JNEUROSCI.3496-07.2007 | No; background gene for network context |
| *gcy-31* | Selected because published literature reported association with pathogen avoidance, sensory behavior, avoidance-related neuronal regulation, neuroimmune regulation, or stress/defense pathways. | Hallem et al., Current Biology, 2011. “Receptor-Type Guanylate Cyclase Is Required for Carbon Dioxide Sensation by *Caenorhabditis elegans*.” DOI: 10.1016/j.cub.2010.12.034 | No; background gene for network context |
| *cwn-2* | Selected because it is a Wnt pathway component examined in this study and showed detectable/stronger support for involvement in PA14 avoidance by RNAi, mutant, rescue, epistasis, or expression analysis. | This study | Yes; experimental support |
| *cfz-2* | Selected because it is a Wnt pathway component examined in this study and showed detectable/stronger support for involvement in PA14 avoidance by RNAi, mutant, rescue, epistasis, or expression analysis. | This study | Yes; experimental support |
| *dsh-1* | Selected because it is a Wnt pathway component examined in this study and showed detectable/stronger support for involvement in PA14 avoidance by RNAi, mutant, rescue, epistasis, or expression analysis. | This study | Yes; experimental support |
| *gsk-3* | Selected because it is a Wnt pathway component examined in this study and showed detectable/stronger support for involvement in PA14 avoidance by RNAi, mutant, rescue, epistasis, or expression analysis. | This study | Yes; experimental support |
| *bar-1* | Selected because it is a Wnt pathway component examined in this study and showed detectable/stronger support for involvement in PA14 avoidance by RNAi, mutant, rescue, epistasis, or expression analysis. | This study | Yes; experimental support |
| *mom-2* | Selected because it was examined as a Wnt-associated gene in the RNAi/screening or validation workflow; included as a tested Wnt component or negative/specificity control. | This study | Yes; screened/tested |
| *mig-1* | Selected because it was examined as a Wnt-associated gene in the RNAi/screening or validation workflow; included as a tested Wnt component or negative/specificity control. | This study | Yes; screened/tested |
| *dsh-2* | Selected because it was examined as a Wnt-associated gene in the RNAi/screening or validation workflow; included as a tested Wnt component or negative/specificity control. | This study | Yes; screened/tested |
| *sys-1* | Selected because it was examined as a Wnt-associated gene in the RNAi/screening or validation workflow; included as a tested Wnt component or negative/specificity control. | This study | Yes; screened/tested |
| *wrm-1* | Selected because it was examined as a Wnt-associated gene in the RNAi/screening or validation workflow; included as a tested Wnt component or negative/specificity control. | This study | Yes; screened/tested |
| *apr-1* | Selected because it was examined as a Wnt-associated gene in the RNAi/screening or validation workflow; included as a tested Wnt component or negative/specificity control. | This study | Yes; screened/tested |
| *mom-1* | Selected because it was examined as a Wnt-associated gene in the RNAi/screening or validation workflow; included as a tested Wnt component or negative/specificity control. | This study | Yes; screened/tested |
| *lin-17* | Selected based on published Wnt signaling annotation and reported Frizzled receptor function in *C. elegans* | Sawa et al., Genes & Development, 1996. “The *Caenorhabditis elegans* gene lin-17, which is required for certain asymmetric cell divisions, encodes a putative seven-transmembrane protein similar to the Drosophila Frizzled protein.” DOI: 10.1101/gad.10.17.2189 | No; pathway-annotation background gene |
| *mom-5* | Selected based on published Wnt signaling studies or pathway annotations to provide Wnt network context; not functionally validated in this study. | Park et al., Current Biology, 2004. “*C. elegans* MOM-5/frizzled functions in MOM-2/Wnt-independent cell polarity and is localized asymmetrically prior to cell division.” DOI: 10.1016/j.cub.2004.12.019 | No; pathway-annotation background gene |
| *hmp-2* | Selected based on published annotation as a *C. elegans* β-catenin-related protein | Korswagen et al., Nature, 2000. “Distinct beta-catenins mediate adhesion and signalling functions in *C. elegans*.” DOI: 10.1038/35020099 | No; pathway-annotation background gene |
| *egl-20* | Selected because it was examined as a Wnt ligand/pathway component in the RNAi or mutant screening workflow. | This study | Yes; screened/tested |
| *cwn-1* | Selected based on published Wnt signaling studies or pathway annotations to provide Wnt network context; not functionally validated in this study. | Zinovyeva et al., Genetics, 2008. “Complex Network of Wnt Signaling Regulates Neuronal Migrations During *Caenorhabditis elegans* Development.” DOI: 10.1534/genetics.108.090290 | No; pathway-annotation background gene |
| *cam-1* | Selected based on published Wnt signaling annotation and reported Ror receptor function in Wnt-mediated cell migration/polarity | Forrester et al., Genetics, 2004. “The *Caenorhabditis elegans* Ror RTK CAM-1 Inhibits EGL-20/Wnt Signaling in Cell Migration.” DOI: 10.1534/genetics.104.031781 | No; pathway-annotation background gene |
| *pop-1* | Selected based on published Wnt signaling annotation and reported POP-1/TCF function in canonical and noncanonical Wnt pathways | Herman, Development, 2001. “*C. elegans* POP-1/TCF functions in a canonical Wnt pathway that controls cell migration and in a noncanonical Wnt pathway that controls cell polarity.” DOI: 10.1242/dev.128.4.581 | No; pathway-annotation background gene |
| *lin-18* | Selected based on published Wnt signaling annotation and reported Ryk receptor function in parallel to LIN-17/Frizzled in Wnt signaling | Inoue et al., Cell, 2004. “*C. elegans* LIN-18 is a Ryk ortholog and functions in parallel to LIN-17/Frizzled in Wnt signaling.” DOI: 10.1016/j.cell.2004.09.001 | No; pathway-annotation background gene |

Note: Genes listed in this table were used to define the predefined input gene set for STRING-based PPI analysis. Literature-curated genes were included to provide avoidance-related, sensory, neuroimmune, or stress-response network context; Wnt-related genes were included as tested components in this study or as pathway-annotation background genes. Inclusion in this table does not indicate direct functional validation in PA14 avoidance unless marked as “Yes; experimental support” in the “Included in this study” column. This table lists the predefined input genes, simplified inclusion basis, supporting references, and whether each gene was experimentally examined in this study. All listed genes were used for STRING-based PPI analysis and displayed in Figure 6E.
